# Multi-omics analysis of N6-methyladenosine reader IGF2BP3 as a promising biomarker in pan-cancer

**DOI:** 10.3389/fimmu.2023.1071675

**Published:** 2023-01-25

**Authors:** Pin Chen, Jing Xu, Zihan Cui, Silin Wu, Tao Xie, Xiaobiao Zhang

**Affiliations:** ^1^ Department of Neurosurgery, Zhongshan Hospital, Fudan University, Shanghai, China; ^2^ Department of Endocrinology and Metabolism, Zhongshan Hospital, Fudan University, Shanghai, China; ^3^ Department of Thoracic Surgery, The First Affiliated Hospital of Soochow University, Medical College of Soochow University, Suzhou, China; ^4^ Institute of Thoracic Surgery, The First Affiliated Hospital of Soochow University, Suzhou, China; ^5^ Cancer Center, Shanghai Zhongshan Hospital, Fudan University, Shanghai, China; ^6^ Digital Medical Research Center, Fudan University, Shanghai, China

**Keywords:** insulin-like growth factor 2 mRNA-binding protein 3 (IGF2BP3), pan-cancer analysis, genetic alteration, prognosis, the Cancer Genome Atlas (TCGA), immune infiltration

## Abstract

**Background:**

Insulin-like growth factor 2 mRNA-binding protein 3 (IGF2BP3) has been reported to exhibit an oncogenic effect as an RNA-binding protein (RBP) by promoting tumor cell proliferation, migration and invasion in several tumor types. However, a pan-cancer analysis of IGF2BP3 is not currently available, and the exact roles of IGF2BP3 in prognosis and immunology in cancer patients remain enigmatic. The main aim of this study was to provide visualization of the systemic prognostic landscape of IGF2BP3 in pan-cancer and to uncover the potential relationship between IGF2BP3 expression in the tumor microenvironment and immune infiltration profile.

**Methods:**

Raw data on IGF2BP3 expression were obtained from GTEx, CCLE, TCGA, and HPA data portals. We have investigated the expression patterns, diagnostic and prognostic significance, mutation landscapes, functional analysis, and functional states of IGF2BP3 utilizing multiple databases, including HPA, TISIDB, cBioPortal, GeneMANIA, GESA, and CancerSEA. Moreover, the relationship of IGF2BP3 expression with immune infiltrates, TMB, MSI and immune-related genes was evaluated in pan-cancer. IGF2BP3 with drug sensitivity analysis was performed from the CellMiner database. Furthermore, the expression of IGF2BP3 in different grades of glioma was detected by immunohistochemical staining and western blot.

**Results:**

We found that IGF2BP3 was ubiquitously highly expressed in pan-cancer and significantly correlated with diagnosis, prognosis, TMB, MSI, and drug sensitivity in various types of cancer. Besides, IGF2BP3 was involved in many cancer pathways and varied in different immune and molecular subtypes of cancers. Additionally, IGF2BP3 is critically associated with genetic markers of immunomodulators in various cancers. Finally, we validated that IGF2BP3 protein expression was significantly higher in glioma than in normal tissue, especially in GBM.

**Conclusions:**

IGF2BP3 may be a potential molecular biomarker for diagnosis and prognosis in pan-cancer, especially for glioma. It could become a novel therapeutic target for various cancers.

## Introduction

The N^6^ adenosine methylation (m6A) is methylated at the N6 site of adenosine and thought to be a dynamic modification of mRNA in mammalian cells (1–3). Distinct from DNA methylation and histone modification is playing a role at the transcriptional level, the m6A modification functions at a post-transcriptional level. Specifically, the m6A modifications achieve the control of the target gene expression through the coordination of 3 classes of regulators, including m6A methyltransferases (‘writers’), m6A modified binding proteins (‘readers’), and m6A demethylase (‘erasers’) (4). In mammals, the m6A ‘writer’ complex mainly contains methyltransferase-like protein 3 (METTL3), methyltransferase-like 14 (METTL14), Wilms-tumour associated protein (WTAP), which catalyzes the m6A modification of adenosine on RNA. Conversely, the m6A erasers mainly consists of fat mass, obesity-associated protein (FTO) and AlkB homolog 5 (ALKBH5) demethylases, which are responsible for removing the m6A marks selectively. Therefore, the m6A modification process is highly dynamic and reversible. The m6A ‘readers’ proteins (such as YTH, IGF2BP, and HNRNP families) are preferentially bind to the m6A-modified mRNA (also called the RNA Binding Proteins, RBPs) and regulate RNA metabolism by serving as readers. Among various readers, the Insulin-like growth factor 2 mRNA-binding proteins (IGF2BPs) including IGF2BP1/2/3 was first identified in 2018. As an essential m6A reader, the stability of target mRNA can be enhanced by modification of m6A (5, 6).

IGF2BP3, a member belonging to the conserved IGF2BP family is highly expressed during both embryogenesis and carcinogenesis and lowly expressed in tissues of healthy adults (7, 8). IGF2BP3 has demonstrated to the malignant transformation of tumor. It includes proliferation, invasion, migration, and drug resistance (9–15). Besides its role as a newly reported m6A reader, IGF2BP3 has also been well-proven to function in cancer metabolism, immunity, angiogenesis, stemness, and differentiation (16–21). Specifically, previous evidence has indicated that IGF2BP3 plays a crucial role in human cancer development, such as breast cancer (10, 22), mesothelioma (11), colon cancer (15, 19), lung cancer (18), melanoma (13), nasopharyngeal carcinoma (NPC) (14), and hepatocellular carcinoma (HCC) (20). Nevertheless, there is still a lack of comprehensive and systematic studies assessing the impact of IGF2BP3 on multiple cancer types.

Recently, pan-cancer analysis of tumorigenesis and progression has become a research focus. Therefore, it is of importance to further investigate the oncogene profile using a pan-cancer strategy. However, there are still no relevant articles on IGF2BP3 and pan-cancer. Here, we performed comprehensive research on the roles of IGF2BP3 in human pan-cancer. Our findings showed that IGF2BP3 expression was significantly higher in most tumors than in adjacent paired normal tissues. Besides, both the diagnostic utility and predictive value of IGF2BP3 in the pan-cancer TCGA cohorts were evaluated. IGF2BP3 genetic alternations were identified using the cBioPortal database. Additionally, we investigated the potential relationship between IGF2BP3 mRNA expression level and clinicopathologic characteristics, tumor mutation burden (TMB), microsatellite instability (MSI), and infiltrating immune cells in pan-cancer. Drug sensitivity analysis of IGF2BP3 was also performed *via* the CellMiner database.

We concluded that IGF2BP3 could serve as a candidate prognostic factor across diverse tumor types. IGF2BP3 exerted its function *via* the regulation of TMB, MSI, tumor immune microenvironment (TME), and drug sensitivity. This study highlights the manifold roles of IGF2BP3 in pan-cancer, which is promising as a prospective biomarker and potential target for cancer therapy.

## Materials and methods

### Data collection and software availability

IGF2BP3 gene expression data and clinical profiles of tumors and their corresponding normal samples were acquired from The Cancer Genome Atlas (TCGA) database (https://portal.gdc.cancer.gov/) and gene type-tissue expression (GTEx) using UCSC Xena (https://xena.ucsc.edu/) (23). Multidimensional analysis of IGF2BP3 expression in different cancer cell lines using the Cancer Cell Line Encyclopedia (CCLE) database (https://portals.broadinstitute.org/) (24). The expression level of IGF2BP3 across human cancer tissues and normal tissues (such as liver, lung, and stomach), as well as the corresponding 24 tumor cell lines (such as liver, thyroid, and lung) was systematically analyzed. The RNA-seq data in TPM format were converted into log2 format for expression comparison between samples (ns, p ≥ 0.05; *p < 0.05; **p < 0.01; ***p < 0.001; ****p < 0.0001).

### Protein level analysis

The Human Protein Atlas (HPA) (http://www.proteinatlas.org/) is a milestone protein research database that contains protein expression in both tumor and normal tissues and is used to probe the protein levels of IGF2BP3. IHC Images of IGF2BP3 protein expression in normal and tumor tissues were downloaded from HPA, including brain, lung, pancreas, colon, cervix, nasopharynx and ovary. The antibody for IHC used was HPA076951.

### IGF2BP3 expression in immune and molecular subtypes of cancers

The correlations between IGF2BP3 expression and immune or molecular subtypes were explored through the TISIDB database (25), an integrated database with a diversity of data types to evaluate tumor-immune system interactions. The association between IGF2BP3 expression and immunomodulators in pan-cancer was also explored based on the TISIDB database.

### Specimen collection

Twenty-two glioma samples were provided by the Department of Neurosurgery, Zhongshan Hospital of Fudan University (Shanghai, China). The three normal tissues surrounding the tumor were normal brain tissues obtained by cortical resection during resection of deep brain glioma. All patients did not receive preoperative chemotherapy or radiotherapy. Tissue samples were extracted and immediately frozen in liquid nitrogen or formalin-fixed. All human samples were used only for research purposes. This study was approved by the Ethics Committee of Zhongshan Hospital, Fudan University.

### Diagnostic value analysis

The subject operating characteristic (ROC) curve was established to assess the diagnostic performance of IGF2BP3 in pan-cancer. The area under the curve was taken to be in the range of 0.5 to 1, with higher values indicating a better diagnostic effect. An AUC value of 0.5–0.7 suggests poor diagnostic efficacy, 0.7–0.9 represents moderate accuracy, and above 0.9 indicates high diagnostic accuracy.

### Survival prognosis analysis

Kaplan–Meier (KM) curve analysis were applied to estimate the association between IGF2BP3 expression and inter-tumor prognosis (OS, DSS, PFI). Next, we explored the relationship between IGF2BP3 expression and prognostic values (OS, DSS and PFI) in different clinical GBMLGG subgroups. The survival package was used for statistical analysis, and the”survminer” package for data visualization.

### Association of IGF2BP3 expression with different clinical features of glioma

The IGF2BP3 gene expression levels in glioma patients with different clinicopathological features are shown by box plots and tables. Gene expression (RNAseq) and corresponding clinical information were extracted from the TCGA database, transformed into transcripts per million reads (TPM) format, and analyzed by log2-transformation. The Wilcoxon rank sum test was applied to compute the data of two groups, and p < 0.05 was considered to a statistically significant difference (ns, p ≥ 0.05; ∗p < 0.05; ∗∗p < 0.01; ∗∗∗p < 0.001).

### Univariate and multivariate Cox regression analyses in glioma

Survival information of overall survival (OS), disease-specific survival (DSS), and progression-free interval (PFI) was downloaded from TCGA database to display the relationship between IGF2BP3 expression and patient outcomes. The median expression of IGF2BP3 within each tumor type was used as a cut-off value to distinguish low- and high-expression subgroups. The univariate survival analysis was performed to analyze the hazard ratio (HR) and 95% confidence intervals (95% C.I.). A hazard ratio (HR) <1 suggests that IGF2BP3 is a beneficial prognostic factor, while HR >1 indicates that IGF2BP3 is a risk factor for survival. Univariate and multifactorial Cox regression analyses of IGF2BP3 and clinical features were undertaken to ascertain their prognostic value in OS, DSS and PFI in GBMLGG. A survival kit was utilized for survival analysis.

### Genetic alteration analysis

The cBioCancer for Cancer Genomics (cBioPortal) (www.cbioportal.org) was utilized to investigate genomic alteration analysis of specific genes (26, 27). In this study, we applied the “Cancer Types Summary” and below “Cancer Type” button for visualizing genomic alterations of IGF2BP3 among cancers from TCGA database. The frequency of IGF2BP3 copy number alterations and mutations in all TCGA tumors was examined, and the results are shown as plotted bar plots.

### Tumor mutation burden, microsatellite instability

Tumor mutational burden (TMB) and microsatellite instability (MSI) have been characterized as the key biological markers of TME (28–31). Spearman’s correlation coefficient was employed to analyze the relationship between IGF2BP3 expression and TMB and MSI.

### Tumor microenvironment

Estimation of stromal and immune cell components in malignant tumor tissues by differences in expression data (ESTIMATE) is a method for calculating stromal or immune scores, represented by the abundance of the immune and stromal components, respectively (32). The higher the score, the greater the proportion of the corresponding component in the TME. The ESTIMATE score is the sum of the stroma score and the immune score, suggesting the combined proportion of both in the TME. IGF2BP3 expression levels and ImmuneScore and StromalScore were acquired for each tumor by “estimate” R package and Spearman correlation analysis. Immune cell infiltration correlation analysis was performed *via* the TIMER2 database (http://timer.cistrome.org) (33).

### Single-cell functional analysis

The functional status of IGF2BP3 in various cancers was studied using CancerSEA (http://biocc.hrbmu.edu.cn/CancerSEA/) (34), a database that can be used to assess the integrated functional status of diverse tumor cells at the single-cell level. In this study, we explored the average correlation of IGF2BP3 with functional status in 18 cancers, including angiogenesis, proliferation, apoptosis, cell cycle, DNA damage, DNA repair, inflammation, hypoxia, epithelial-mesenchymal transition (EMT), invasion, metastasis, differentiation, quiescence, and stemness. The threshold of IGF2BP3 associated with each tumor functional status was established as a threshold value of |r| >0.3 and a discrimination significance (p < 0.05).

### Protein–protein interaction network and enrichment analysis

GeneMANIA (http://www.genemania.org) is an interactive and flexible online tool for building and visualizing protein-protein interaction (PPI) networks using bioinformatics methods such as physical interaction, co-expression, co-localization, gene enrichment analysis, gene interaction and site prediction, including generating reasonable hypotheses about gene function prediction and detecting Genes that share similar functions (35, 36). In this study, GeneMANIA was employed for PPI analysis of IGF2BP3. Gene set enrichment analysis (GSEA) was used to detect the IGF2BP3 affected pathway in tumors. The entire biological process is assessed on the basis of the Kyoto Encyclopedia of Genes and Genomes (KEGG) and HALLMARK pathways.

### Drug sensitivity of IGF2BP3 in pan-cancer

NCI-60 compound activity data and RNA-seq expression profiles from the CellMiner™ were downloaded to analyze the drug sensitivity of IGF2BP3 in pan-cancer (https://discover.nci.nih.gov/cellminer/home.do) (37). Drugs approved by FDA or clinical trials were selected for analysis.

### Immunohistochemsitry

Tissues were formalin-fixed and paraffin-embedded and sectioned to 4 mm layer thickness regularly. Tissue sections were processed and stained with the following antibodies: IGF2BP3 (1:300, 14642-1-AP, Proteintech).

### Western blot analysis

Total protein was isolated from tissues and quantified with the BCA protein quantification kit (Beyotime, #P001). Equal amounts of proteins separated by 10% SDS-PAGE, transferred to polyvinylidene fluoride membranes (0.45 μM PVDF, Millipore, USA), then the membranes were blocked with skimmed milk for 1 hour and incubated with primary antibody IGF2BP3 (1:1000, 14642-1-AP, Proteintech) overnight at 4°C. The corresponding HRP-conjugated secondary antibody (#A0208,1:2000, Beyotime Biotechnology, Shanghai, China) used, and the bands visualized by ECL Western blotting substrate (Thermo Fisher Scientific, USA). The intensity of protein expression was detected *via* ImageJ software.

## Results

### Expression and mutant aspects of IGF2BP3 in pan-cancer

**Figure 1:**
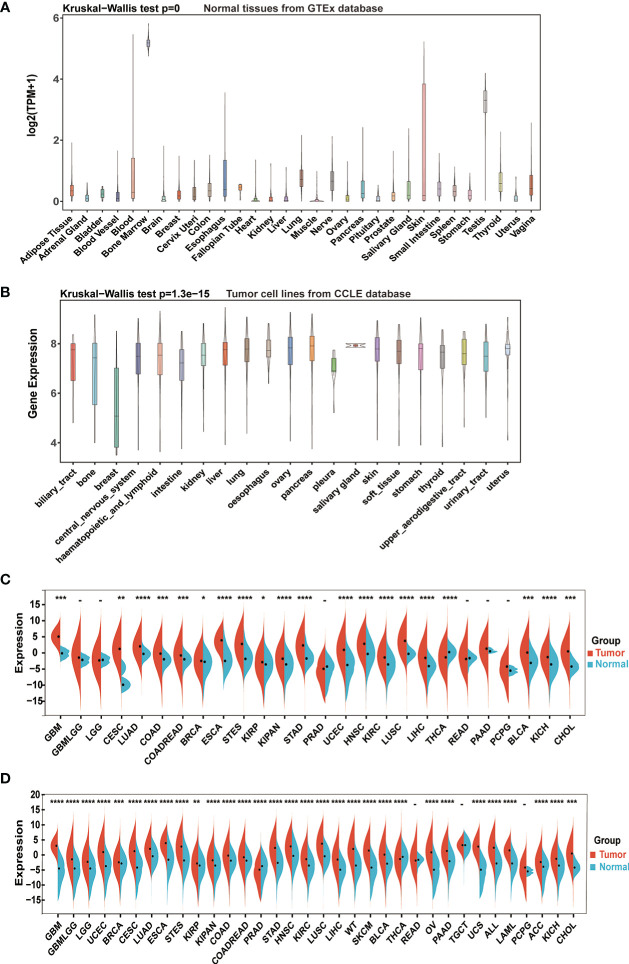
IGF2BP3 mRNA expression levels in pan-cancer. **(A)** IGF2BP3 expression levels in normal tissues from GTEx database. **(B)** IGF2BP3 expression levels in tumor cell lines from CCLE database. **(C)** IGF2BP3 expression levels in tumor tissues from TCGA database. **(D)** IGF2BP3 expression difference between tumor tissues from TCGA database and normal tissues from the GTEx database; ns, no significance; *p < 0.05, **p < 0.01, ***p < 0.001, and ****p < 0.0001.

The study flowchart is illustrated in **Supplementary Figure 1**. First, we assessed IGF2BP3 mRNA levels in normal human tissues, using the GTEx dataset. As shown in **Figure 1A**, the IGF2BP3 level varied across multiple types of tissue was remarkably high in bone marrow (BM). BM is known to be a highly differentiating tissue, and higher expression levels are not entirely unexpected. In addition, we examined the expression levels of IGF2BP3 across various tumor types. In different cancer cell lines from the CCLE database, not only were IGF2BP3 expression levels significantly and generally elevated but smaller ranges were shown compared to the range of expression in normal human tissues (**Figure 1B**).

Further comparison between the tumors and adjacent normal tissues displayed that the expression level of IGF2BP3 was upregulated in most types of human cancers. Directly, considering TCGA data alone, the gene expression difference achieved considerable significance in 20 of 26 TCGA cancer types, with the exception of glioma (GBMLGG), brain lower grade glioma (LGG), prostate adenocarcinoma (PRAD), rectum adenocarcinoma (READ), pancreatic adenocarcinoma (PAAD) and pheochromocytoma and paraganglioma (PCPG). Moreover, only in thyroid carcinoma (THCA) IGF2BP3 had an increased expression in corresponding normal tissues instead of tumor samples, which was the opposite of the condition in other cancer types (**Figure 1C**).

To further compare IGF2BP3 expression between the tumor and normal tissues, we combined data from TCGA and GTEx. Results from combined databases revealed that IGF2BP3 was over-expressed significantly in 31 out of 34 cancer types (exceptions were READ, TGCT, and PCPG). Mainly, IGF2BP3 was highly expressed in diverse tumor types, such as GBMLGG, glioblastoma multiforme (GBM), LGG, lung adenocarcinoma (LUAD), lung squamous cell carcinoma (LUSC), PAAD, head and neck squamous cell carcinoma (HNSC), uterine corpus endometrial carcinoma (UCEC), colon adenocarcinoma (COAD), and esophageal carcinoma (ESCA). However, reversed results with significance were observed in PRAD and THCA (**Figure 1D**).

**Figure 2:**
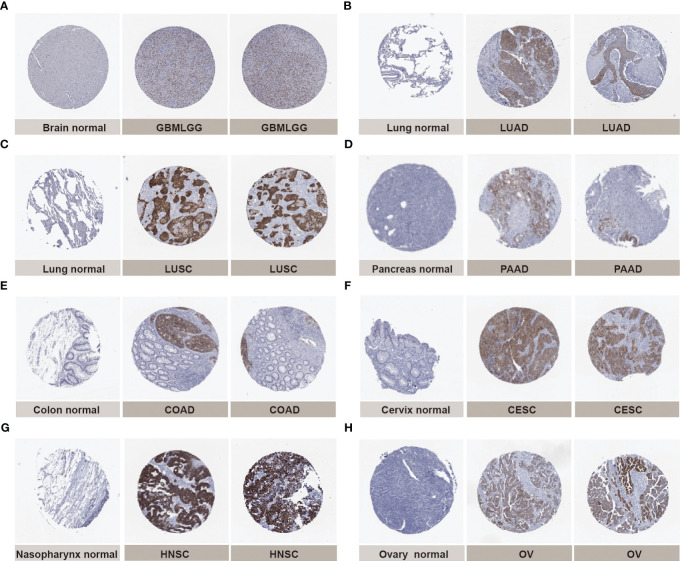
Representative immunohistochemical staining (IHC) in multiple normal (left) and tumor (right) tissues. The protein expression of IGF2BP3 in **(A)** glioma, GBMLGG; **(B)** lung adenocarcinoma, LUAD;**(C)** lung squamous cell carcinoma, LUSC; **(D)** pancreatic adenocarcinoma, PAAD; **(E)** colon adenocarcinoma, COAD; **(F)** cervical squamous cell carcinoma and endocervical adenocarcinoma, CESC; **(G)** head and neck squamous cell carcinoma, HNSC; **(H)** ovarian serous cystadenocarcinoma, OV.

Next, we verified the expression of IGF2BP3 between cancer tissues and adjacent normal tissues at protein level using the HPA database. Compared to weak IHC positive staining in normal brain, lung, pancreas, colon, cervix, nasopharynx, and ovary tissues, much stronger staining of IGF2BP3 was examined in GBMLGG, LUAD, LUSC, PAAD, COAD, cervical squamous cell carcinoma and endocervical adenocarcinoma (CESC), HNSC, and ovarian serous cystadenocarcinoma (OV) tissues in terms of protein level (**Figures 2A–H**). The results from the two databases (TCGA and HPA) were broadly consistent.

**Figure 3:**
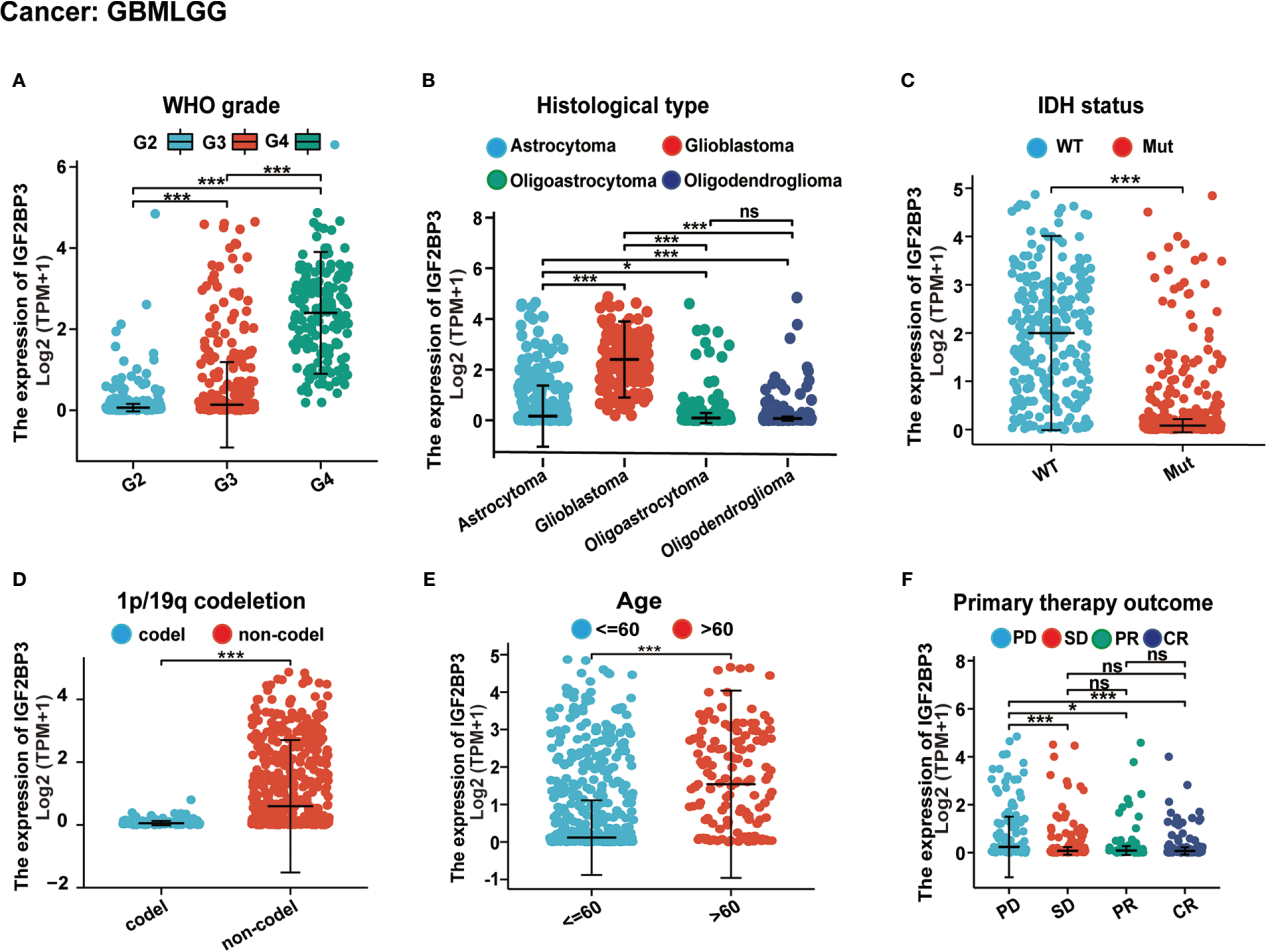
Associations between IGF2BP3 expression and different clinical characteristics in GBMLGG. **(A)** WHO grade; **(B)** Histological type; **(C)** IDH status; **(D)** p/19q codeletion; **(E) **Age; **(F)** Primary therapy outcome. ns, p ≥ 0.05; *p < 0.05; ***p < 0.001.

Further, we assessed the associations between IGF2BP3 and different clinical characteristics in pan-cancer. For GBMLGG, IGF2BP3 expression was significantly correlated with World Health Organization (WHO) grade, histological type, IDH status, 1p/19q codeletion, primary therapy outcome, and age of GBMLGG (**Supplementary Table 1**). Specifically, the expression level of IGF2BP3 increased significantly with increasing WHO grade gliomas (**Figure 3A**). Moreover, IGF2BP3 showed higher levels in patients with GBM in comparison with other histological types of glioma (**Figure 3B**). Next, we subdivided the TCGA patients according to different IDH mutations and 1p/19q codeletion status and found that high IGF2BP3 expression positively correlated with IDH status (wildtype), and 1p/19q non-codeletion (**Figures 3C, D**). Additionally, IGF2BP3 was expressed higher in patients with age >60 (**Figure 3E**), and primary therapy outcome (PD) (**Figure 3F**), respectively.

### Mutation analysis of IGF2BP3

It is well recognized that DNA methylation and genetic alterations are tightly linked to the occurrence and development of tumors. Herein, we initially analyzed the IGF2BP3 alteration status across multiple cancer types using cBioPortal database **(** **Supplementary Figure 2** **)**. Among all cancers tested, the IGF2BP3 gene was amplified in multiple types of cancer, with the highest alteration frequency (>6%) in uterine carcinosarcoma (UCS). Notably, the type of mutation was the primary type in the UCEC, skin cutaneous melanoma (SKCM), stomach adenocarcinoma (STAD), and COAD, which show an alteration frequency of ~4% **(** **Supplementary Figure 2A** **)**. The types, sites and case numbers of the IGF2BP3 gene mutation were further displayed above the bars **(** **Supplementary Figure 2C** **).** Overall, as shown in **Supplementary Figure 2B**, amplification was the main type of alteration, while the most frequent putative copy-number alterations of IGF2BP3 were amplification, gain function, and diploid. Finally, in the present study, the gene alteration of DNAH11, GPNMB, TP53, KLHL7, NUP42, MALSU1, ABCB5, STK31, TRA2A, and HDAC9 was more common in the altered group than in the unaltered group across the cBioPortal database **(** **Supplementary Figure 2D** **)**. As dysregulated IGF2BP3 was implicated in the process of RNA regulation and transcription in cancer, we further investigated whether IGF2BP3 was associated with the mutation of cancer-related genes. Here, we took LGG as an example to illustrate the correlation between the IGF2BP3 expression level and mutation frequencies. As shown in **Supplementary Figure 2E**, in LGG, the top five frequently mutated genes remained as IDH1 (82.6%), CIC (20.6%), TTN (12.8%), MUC16 (7.4%), and EGFR (7.2%). Moreover, the previously mentioned mutated genes with significance defined by FDR < 0.05. These results indicate that the IGF2BP3 is tightly correlated with cancer-related gene mutation status.

### Diagnostic value of IGF2BP3 in pan-cancer

**Figure 4:**
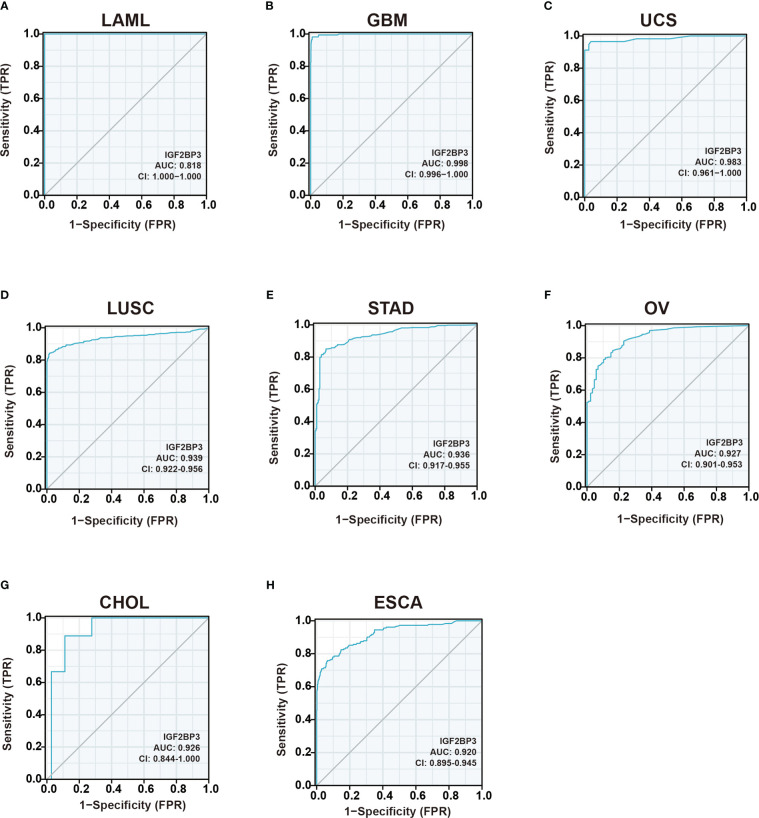
Receiver operating characteristic (ROC) curve for IGF2BP3 expression in pan-cancer.**(A)** LAML; **(B)** GBM; **(C)** UCS; **(D)** LUSC; **(E)** STAD; **(F)** OV; **(G)** CHOL; **(H)** ESCA.

As shown in **Figures 4A–H**, IGF2BP3 has an exact accuracy (AUC > 0.7) in predicting 24 cancer types, and even exceeded 0.9 in 8 cancers including LAML (AUC = 1.0), GBM (AUC = 0.998), UCS (AUC = 0.983), LUSC (AUC = 0.939), STAD (AUC = 0.936), OV (AUC = 0.927), CHOL (AUC = 0.926), and ESCA (AUC = 0.920) (**Supplemental Table 2**), which had high diagnostic value.

### Prognostic value of IGF2BP3 across cancers

**Figure 5:**
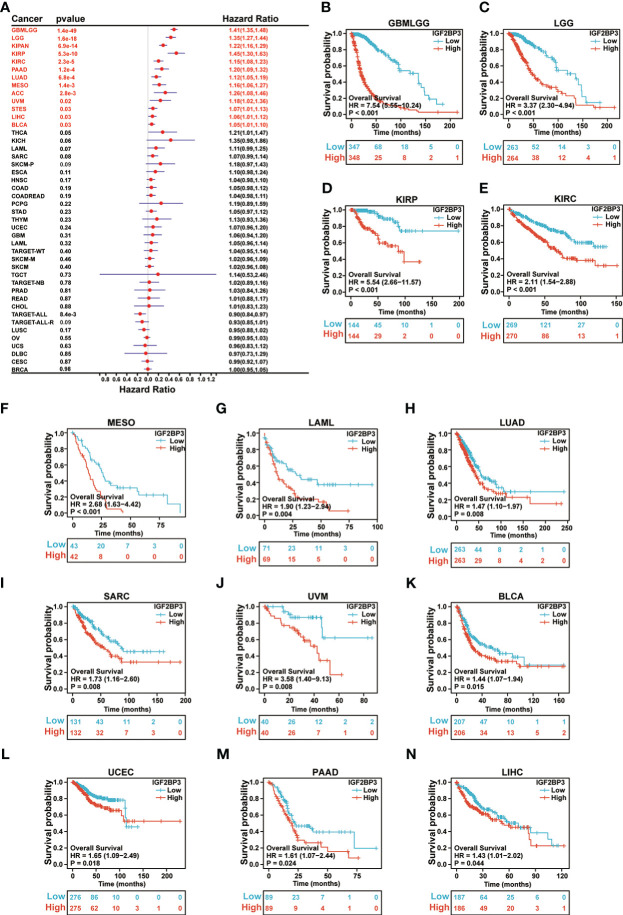
Relationship of IGF2BP3 expression with patient Overall Survival (OS). **(A)** Forest map shows the univariate Cox regression analysis results for IGF2BP3 in TCGA pan-cancer samples. **(B–N)** Kaplan–Meier analysis of the association between IGF2BP3 expression and OS.

Further, each cancer’s survival analysis was performed to investigate the association between IGF2BP3 expression level and prognosis, concentrating on OS, DSS, and PFI. The forest plot of the univariate Cox model suggested that IGF2BP3 was a significant risk factor for OS in GBMLGG (p < 0.001), LGG (p < 0.001), KIPAN (p < 0.001), KIRP (p < 0.001), KIRC (p < 0.001), PAAD (p < 0.001), LUAD (p < 0.001), LAML (p = 0.0011), MESO (p = 0.0014), ACC (p = 0.0028), UVM (p = 0.02), STES (p = 0.03), LIHC (p = 0.03), and BLCA (p = 0.03) patients (**Figure 5A**). Next, the Kaplan-Meier analysis of OS indicated that patients with high expression of IGF2BP3 was significantly correlated with poor prognosis in patients with GBMLGG (p < 0.001), LGG (p < 0.001), KIRP (p < 0.001), KIRC (p < 0.001), MESO (p < 0.001), LAML (p = 0.004), LUAD (p = 0.008), SARC (p = 0.008), UVM (p = 0.008), BLCA (p = 0.015), UCEC (p = 0.018), PAAD (p = 0.024), and LIHC (p = 0.044) (**Figures 5B–N**).

Moreover, as presented in **Supplementary Figure 3A**, we performed Cox regression analysis of DSS and identified that IGF2BP3 was an independent risk factor in patients with GBMLGG (p < 0.001), LGG (p < 0.001), KIPAN (p < 0.001), KIRP (p < 0.001), KIRC (p < 0.001), PAAD (p < 0.001), MESO (p < 0.001), LUAD (p = 0.0013), ACC (p = 0.0017), UVM (p = 0.0079), STES (p = 0.0084), SKCM-P (p = 0.03), and KICH (p = 0.04). Notably, the resulting Kaplan-Meier survival analysis indicated that patients with higher IGF2BP3 expression tended to exhibit a significantly shorter DSS as compared to those with lower IGF2BP3 expression, respectively, in GBMLGG (p < 0.001), LGG (p < 0.001), KIRP (p < 0.001), KIRC (p < 0.001), MESO (P < 0.001), UVM (p = 0.008), SARC (p = 0.009), PAAD (p = 0.01), UCEC (P = 0.018), and LUAD (P = 0.023) (**Supplementary Figures 3B-K**).

Also, univariate Cox regression analysis of PFI analyses was performed, and the results showed that IGF2BP3 was a risk factor in patients with high-risk factor in GBMLGG (p < 0.001), LGG (p < 0.001), KIPAN (p < 0.001), KIRC (p < 0.001), KIRP (p < 0.001), PAAD (p < 0.001), UVM (p < 0.001), LUAD (p = 0.0043), LIHC (p = 0.005), ACC (p = 0.0061), SKCM-P (p = 0.0064), and MESO (p = 0.02) (**Supplementary Figure 4A**). Furthermore, KM plotter analysis revealed that patients with higher IGF2BP3 expression had poorer PFI than those with lower IGF2BP3 expression in GBMLGG (p < 0.001), LGG (p < 0.001), KIRP (p < 0.001), KIRC (p < 0.001), MESO (p < 0.001), UVM (p =0.005), LIHC (p =0.006), and UCEC (p =0.014), as seen in **Supplementary Figures 4B–I**.

**Figure 6:**
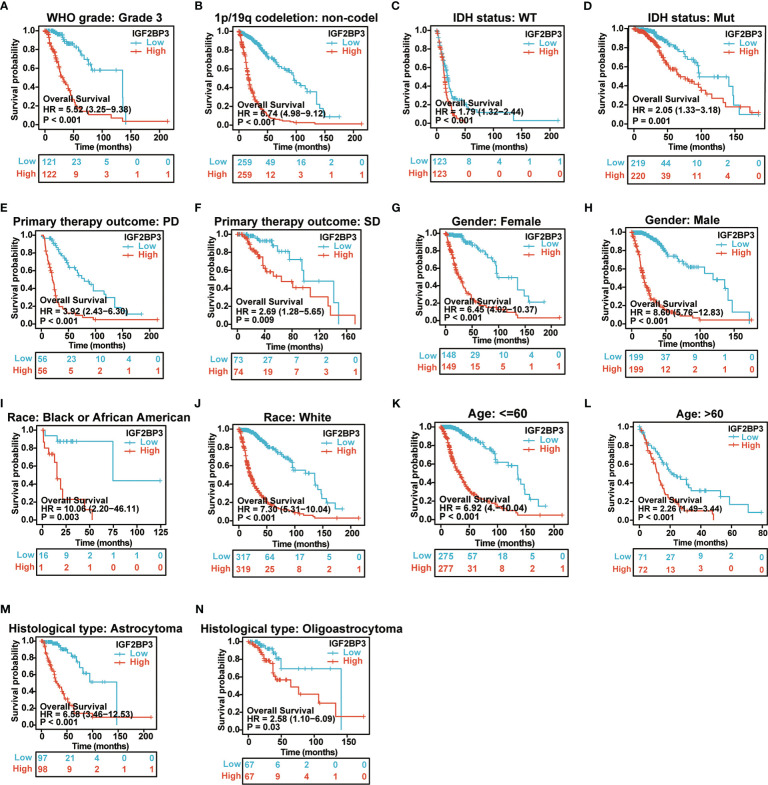
Associations between IGF2BP3 expression and the OS in different clinical subgroups of GBMLGG. **(A)** WHO grade (G3); **(B)** 1p/19q codeletion (non−codel); **(C)** IDH status (WT); **(D)** IDH status (Mut); **(E)** Primary therapy outcome (PD); **(F)** Primary therapy outcome (SD); **(G)** Gender (Female); **(H)** Gender (Male); **(I)** Race (Black or African American) **(J)** Race (White); **(K)** Age ≤ 60; **(L)** Age>60; **(M)** Histological type (Astrocytoma); **(N)** Histological type (Oligoastrocytoma).

We further examined the associations of IGF2BP3 with prognosis (OS, DSS and PFI) in different clinical glioma subgroups. The results of the subgroup analysis demonstrated that high expression of IGF2BP3 was associated with worse OS in most clinical subgroups, including a subgroup of WHO grade: G3 (**Figure 6A**), 1p/19q codeletion: non−codel (**Figure 6B**), a subgroup of IDH status: WT (**Figure 6C**), a subgroup of IDH status: Mut (**Figure 6D**), a subgroup of Primary therapy outcome: PD (**Figure 6E**), a subgroup of Primary therapy outcome: SD (**Figure 6F**), a subgroup of Gender: Female (**Figure 6G**), a subgroup of Gender: Male (**Figure 6H**), a subgroup of Race: Black or African American (**Figure 6I**), subgroup of Race: White (**Figure 6J**), a subgroup of Age: <=60 (**Figure 6K**), a subgroup of Age: >60 (**Figure 6L** **)**, a subgroup of Histological type: Astrocytoma (**Figure 6M**), and Histological type: Oligoastrocytoma (**Figure 6N**).

For DSS, the higher expression of IGF2BP3 had a worse DSS in a subgroup of WHO grade: G3, a subgroup of IDH status: WT, a subgroup of IDH status: Mut, a subgroup of 1p/19q codeletion: non−codel, a subgroup of Primary therapy outcome: PD, a subgroup of Primary therapy outcome: SD, a subgroup of Gender: Female, a subgroup of Gender: Male, a subgroup of Race: Black or African American, a subgroup of Race: White, a subgroup of Age: <=60, a subgroup of Age: >60, a subgroup of Histological type: Astrocytoma, and Histological type: Oligoastrocytoma **(** **Supplementary Figures 5A–M** **)**.

For PFI, the higher expression of IGF2BP3 had a worse PFI in a subgroup of WHO grade: G3, a subgroup of IDH status: WT, a subgroup of IDH status: Mut, a subgroup of 1p/19q codeletion: non−codel, a subgroup of Primary therapy outcome: PD, a subgroup of Gender: Female, a subgroup of Gender: Male, a subgroup of Race: Black or African American, a subgroup of Race: White, a subgroup of Age: <=60, a subgroup of Age: >60, and a subgroup of Histological type: Astrocytoma (**Supplementary Figures 6A–L**).

### Univariate and multivariate Cox regression analyses in GBMLGG patients

Uni- and multivariate Cox regression analyses of IGF2BP3 and clinical characteristics, were performed in TCGA-GBMLGG cohort. In univariate and multivariate Cox regression analyses, age, WHO grade, IDH status, 1p/19q codeletion, primary therapy outcome, histological type, and IGF2BP3 were significantly associated with the OS **(** **Table 1** **)**. In contrast, primary therapy outcome, age, and IGF2BP3 were significantly correlated with DSS (**Supplementary Table 3**), and primary therapy outcome, IDH status, age, and IGF2BP3 were correlated significantly with PFI **(** **Supplementary Table 4** **)**.

**Table 1:** Univariate and multivariate Cox regression analyses of clinical characteristics associated with OS of glioma.

Characteristics	Total (N)	Univariate analysis		Multivariate analysis	
		Hazard ratio (95% CI)	P value	Hazard ratio (95% CI)	P value
**WHO grade**	634				
G2	223	Reference			
G3	243	2.999 (2.007-4.480)	**<0.001**	2.258 (1.452-3.511)	**<0.001**
G4	168	18.615 (12.460-27.812)	**<0.001**	11.151 (3.459-35.948)	**<0.001**
**Age**	674				
<=60	541	Reference			
>60	133	4.500 (3.409-5.940)	**<0.001**	3.929 (2.282-6.764)	**<0.001**
**IDH status**	664				
WT	232	Reference			
Mut	432	0.110 (0.083-0.146)	**<0.001**	0.506 (0.276-0.930)	**0.028**
**1p/19q codeletion**	688				
Codel	170	Reference			
non-codel	518	4.428 (2.885-6.799)	**<0.001**	2.050 (1.224-3.435)	**0.006**
**Primary therapy outcome**	461				
PD	112	Reference			
SD	147	0.440 (0.294-0.658)	**<0.001**	0.425 (0.266-0.680)	**<0.001**
PR	64	0.170 (0.074-0.391)	**<0.001**	0.209 (0.075-0.586)	**0.003**
CR	138	0.133 (0.064-0.278)	**<0.001**	0.143 (0.068-0.302)	**<0.001**
**Histological type**	674				
Astrocytoma	192	Reference			
Glioblastoma	155	6.602 (4.739-9.197)	**<0.001**		
Oligoastrocytoma	132	0.604 (0.374-0.975)	**0.039**	1.117 (0.633-1.970)	0.703
Oligodendroglioma	195	0.543 (0.363-0.813)	**0.003**	0.504 (0.273-0.933)	**0.029**
**IGF2BP3** (High vs. Low)	695	1.776 (1.650-1.910)	**<0.001**	1.539 (1.264-1.875)	**<0.001**

### IGF2BP3 expression in different immune and molecular subtypes of cancers

**Figure 7:**
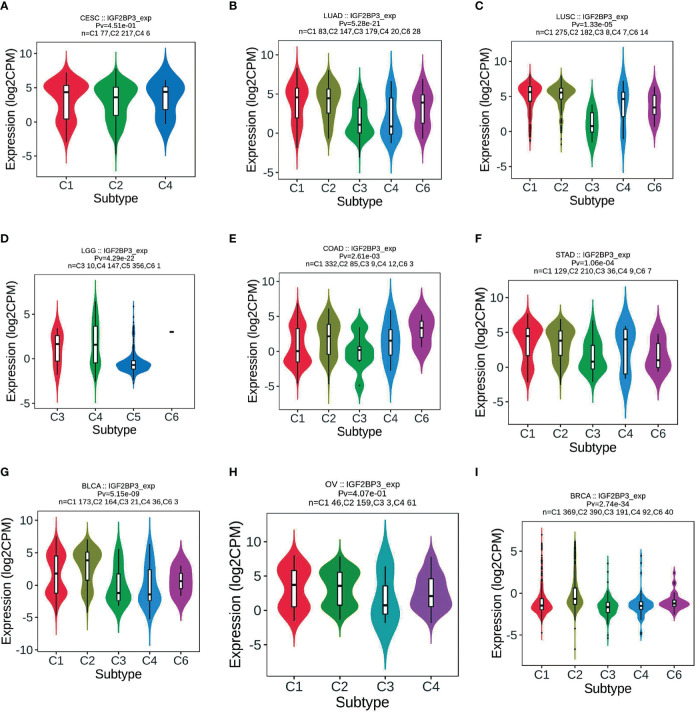
Correlations between IGF2BP3 expression and **immune subtypes** across TCGA tumors. **(A)** CESC; **(B)** LUAD; **(C)** LUSC; **(D)** LGG; **(E)** COAD; **(F)** STAD; **(G)** BLCA; **(H)** OV; **(I)** BRCA.

Correlation of IGF2BP3 differential expression with molecular subtypes in pan-cancer was investigated by the TISIDB database. We found that IGF2BP3 was expressed differently in different immune subtypes (C1: wound healing, C2: IFN-gamma dominant, C3: inflammatory, C4: lymphocyte depleted, C5: immunologically quiet, C6: TGF-b dominant) of 29 cancer types. These include, for example, CESC (**Figure 7A**), LUAD (**Figure 7B**), LUSC (**Figure 7C**), LGG (**Figure 7D**), COAD (**Figure 7E**), STAD (**Figure 7F**), BLCA (**Figure 7G**), OV (**Figure 7H**), and BRCA (**Figure 7I**). In addition, we observed that IGF2BP3 expression was strongly associated with immune stimulators and immune inhibitors (**Supplementary Figure 7**) among nearly all malignancies, represented by UVM, GBMLGG, LGG, LUAD, and KIRC.

**Figure 8:**
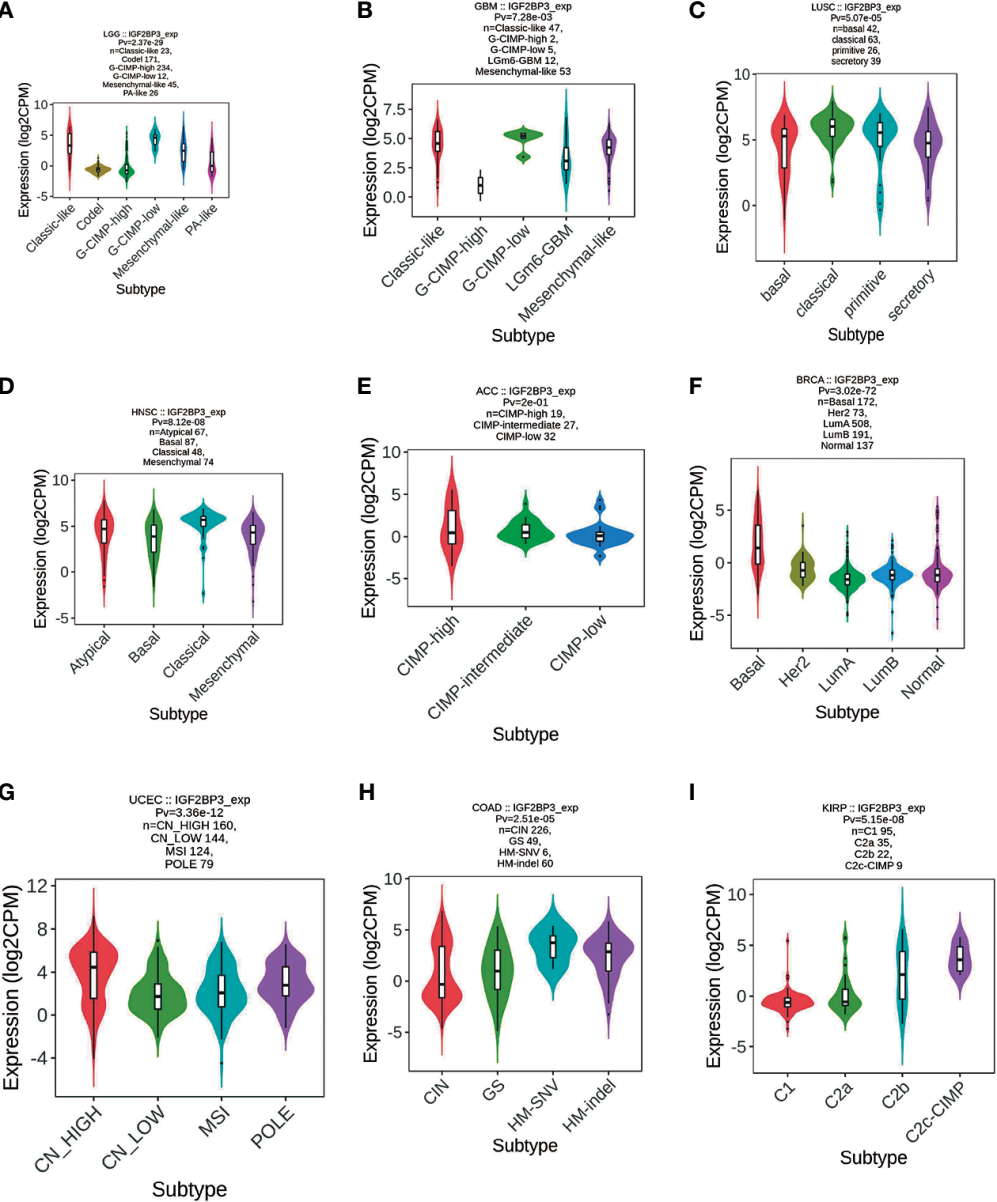
Correlations between IGF2BP3 expression and molecular subtypes across TCGA tumors. **(A)** LGG; **(B)** GBM; **(C)** LUSC; **(D)** HNSC; **(E)** ACC; **(F)** BRCA; **(G)** UCEC; **(H)** COAD; **(I)** KIRP.

Meanwhile, we observed that IGF2BP3 expression was significantly correlated with molecular subtypes of 16 cancer types, such as LGG (**Figure 8A**), GBM (**Figure 8B**), LUSC (**Figure 8C**), HNSC (**Figure 8D**), ACC (**Figure 8E**), BRCA (**Figure 8F**), UCEC (**Figure 8G**), COAD (**Figure 8H**), and KIRP (**Figure 8I** **)**. Further, for LGG and GBM, IGF2BP3 was identified to express the highest in the molecular subtype of G-CIMP-low (**Figures 8A, B**). For LUSC and HNSC, IGF2BP3 was identified to express the highest in the molecular subtype of classical (**Figures 8C, D**). For ACC, IGF2BP3 expression was identified to be the highest in CIMP-intermediate molecular subtype (**Figure 8E**). For COAD, IGF2BP3 was expressed the highest in the molecular subtype of HM-SNV (**Figure 8H**). For STAD, IGF2BP3 was the most highly expressed in the molecular subtype of CIN (**Figure 8E**). For BRCA, IGF2BP3 showed the highest expression in the molecular subtype of basal (**Figure 8F**). For UCEC, IGF2BP3 expression was identified to be the highest in the molecular subtype of CN_HIGH (**Figure 8G**). For KIRP, IGF2BP3 was expressed the highest in the molecular subtype of C2c-CIMP (**Figure 8I**).

### Immune aspects of IGF2BP3 in the tumor microenvironment

**Figure 9:**
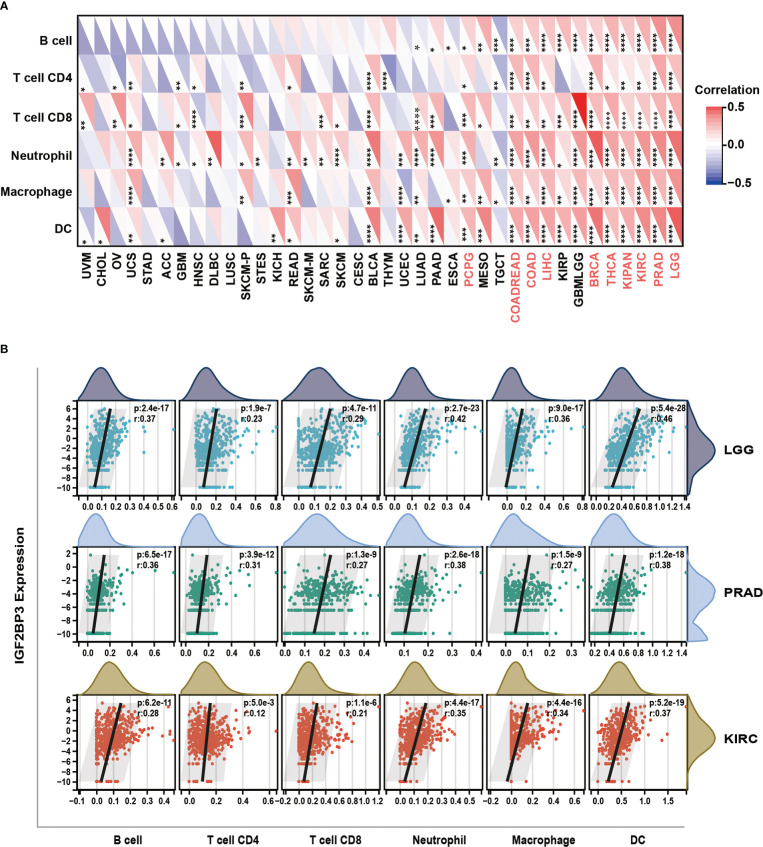
Relationship of IGF2BP3 expression with Immune cell infiltration analysis. **(A)** The relationship between IGF2BP3 expression levels and the levels of infiltration of six immune-related cells based on TIMER database. **(B)** Analysis of immune-associated cells infiltration with IGF2BP3 expression in pan-cancer. p ≥ 0.05; *p < 0.05; **p < 0.01; ***p < 0.001.

We further investigated the relationship between IGF2BP3 expression and immune cell infiltration in pan-cancer levels using immune cell infiltration data extracted from various databases. First, based on the TIMER (Tumor Immune Estimation Resource) database (https://cistrome.shinyapps.io/timer/), we measured six subpopulations of immune cells in TCGA data set including B cells, CD4+ T cells, CD8+ T cells, Neutrophils, Macrophages and Dendritic cells. Generally, as shown in **Figure 9A**, the IGF2BP3 expression had a significantly positive relationship with the infiltration of multiple immune cells, including T cells CD4, T cells CD8, Neutrophil, Macrophages and dendritic cells (DC) in a variety of cancer types. Significantly, some particular cancer types such as LGG, PRAD, and KIRC had a high infiltration level of all three types of immune cells (**Figure 9B**).

**Figure 10:**
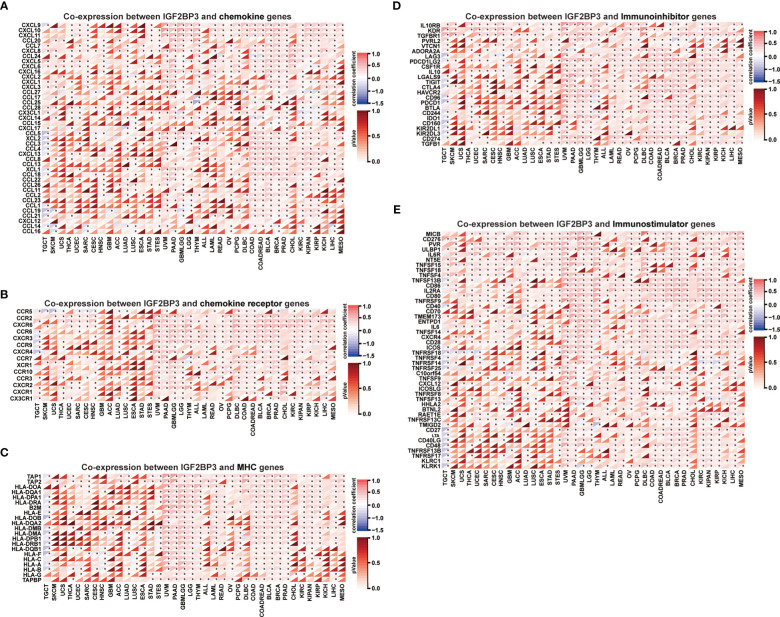
Co-expression of IGF2BP3 and immune-related genes in pan-cancer. Heatmaps indicating the co-expression of IGF2BP3 with immune-relevant genes in pan-cancer, including chemokine genes **(A)**, chemokine-receptor genes **(B)**, MHC molecules **(C)**, immunoinhibitors **(D)**, and immunostimulators **(E)**. *p-value < 0.05, **p-value < 0.01, ***p-value < 0.001, and ****p-value < 0.0001.

Moreover, a co-expression analysis was performed among 33 tumors to investigate the relationships between IGF2BP3 expression and immune-related genes. In accordance with the results (**Figures 10A–E**), there was a strong correlation between IGF2BP3 and most immune-related genes in specific cancer types such as GBMLGG, LGG, LUAD, PAAD, BRCA, and PRAD. Specifically, chemokines such as CXCL9, CXCL10, and CXCL11 and chemokine receptors such as CXCR5, CCR4, CCR8, and CCR1 were positively correlated with IGF2BP3 expression in various cancer types. MHC genes co-expressed with IGF2BP3 in almost all tumor types, particularly in UVM, PAAD, GBMLGG, LGG, KIRC, KIPAN, KIRP, COAD, BLCA, BRCA, PRAD, and LIHC. Moreover, immunostimulatory factors and immunosuppressive factors were also tightly correlated with IGF2BP3 expression in TCGA pan-cancer. Overall, these results show that the expression of IGF2BP3 is closely linked to the biological function of various cytokines and immune-relevant genes.

As it is well known, TMB and MSI in the tumor microenvironment are the most important biomarkers for predicting the therapeutic efficacy of tumor immunotherapy in various tumor types. Outcomes from several studies indicated that tumors with high TMB/MSI status considered to manifest better responses to immunotherapy than those with low TMB/MSI. Thus, we evaluated the correlation between the IGF2BP3 gene expression and TMB and MSI in pan-cancer. As can be seen in **Supplementary Figure 8A**, a significant correlation (P<0.05) existed between IGF2BP3 expression and TMB in 14 categories of cancer. Specifically, IGF2BP3 expression was positively correlated with TMB in LGG, LUAD, LUSC, PRAD, BLCA, PAAD, SARC, BRCA, COAD, SKCM, KIRC, HNSC, and ACC while negatively correlated with TMB only in THCA. Further, we found that the expression of IGF2BP3 was positively related to the MSI in 6 cancers, including LUSC, BLCA, TGCT, ESCA, SARC, and COAD, but had a negative correlation with MSI in SKCM, THCA, HNSC, and DLBC (**Supplementary Figure 8B**).

### Functional states of IGF2BP3 in scRNA-Seq datasets

To evaluate the functional state of IGF2BP3 in various cancer types at the single-cell level, we analyzed the correlation of IGF2BP3 with multiple functional states of cancer cells *via* the CancerSEA. This cancer’s single-cell state atlas revealed a positive correlation of IGF2BP3 with angiogenesis, differentiation, inflammation, metastasis, and quiescence. Negative correlations were observed between IGF2BP3 expression and apoptosis, cell cycle, DNA damage, and DNA repair (**Supplementary Figure 9A**). We then explored the correlation between IGF2BP3 and the functional state in specific cancers. The results found that IGF2BP3 positively correlated with cell cycle and DNA damage in GBM; with metastasis in Astrocytoma; with metastasis, angiogenesis, quiescence, and differentiation in LUAD; with stemness and DNA damage in NSCLC; with angiogenesis, differentiation, and inflammation in RB; with invasion in AML. Conversely, the IGF2BP3 was negatively correlated with cell cycle and DNA damage in Glioma, apoptosis in NSCLC, DNA repair, cell cycle, and DNA damage in RB, angiogenesis in AML, DNA repair, DNA damage, apoptosis, and differentiation in UM (**Supplementary Figures 9B–I**).

### PPI network of IGF2BP3 in cancers and enrichment analysis

**Figure 11:**
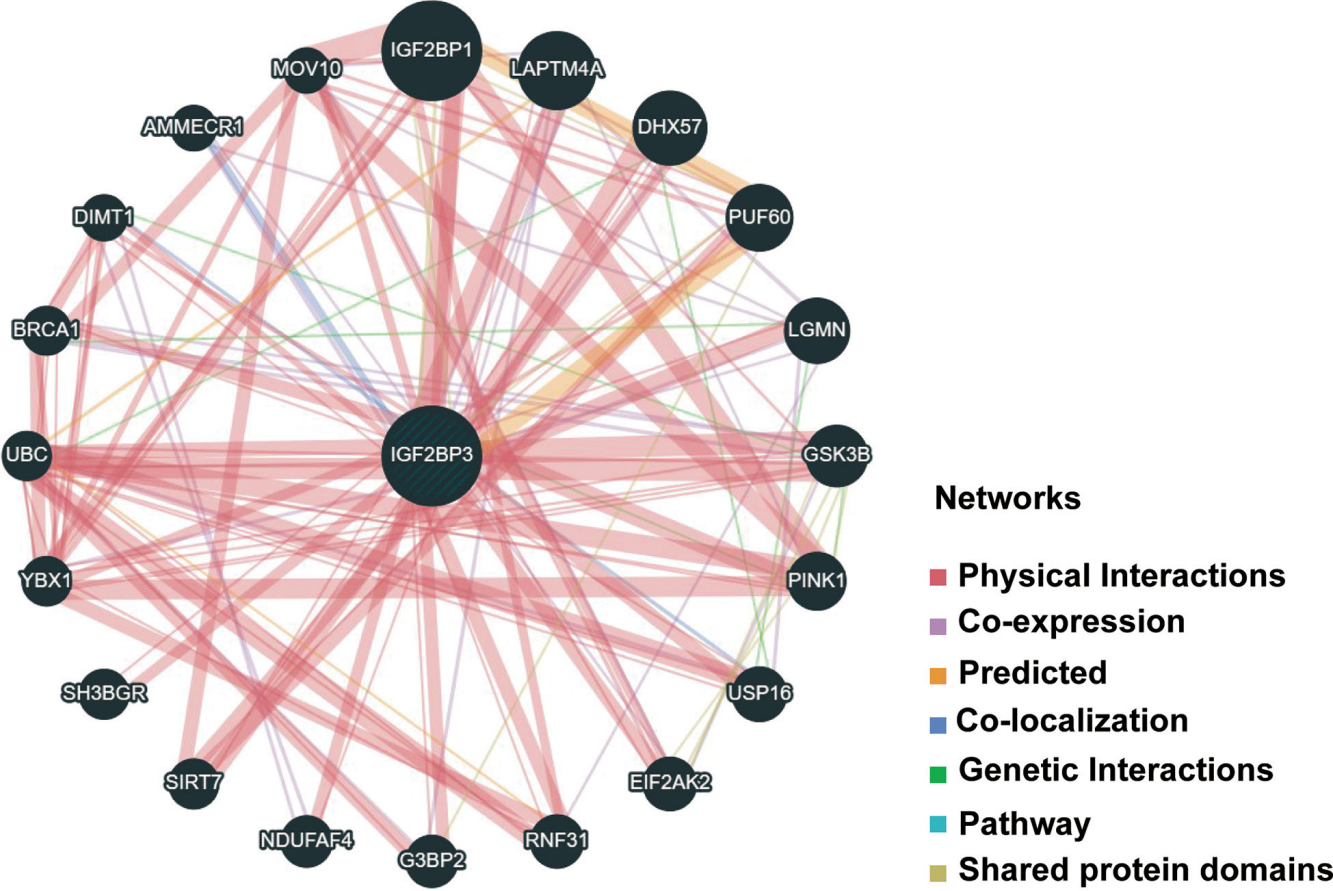
PPI network for IGF2BP3 was constructed *via* GeneMANIA. Different colors of the network edge indicate the bioinformatics methods applied: physical interaction, coexpression, predicted, colocalization, pathway, genetic interaction, and shared protein domains. PPI, protein–protein interaction.

Next, functional network was constructed through GeneMANIA database to explore the potential interactome with IGF2BP3 protein as hub, and the result is shown in **Figure 11**. As evident in the figure, IGF2BP3 had strong physical interactions with IGF2BP1, which are both conserved IGF2BPs predominantly expressed during embryonic development but comparatively lower or silenced in adulthood (5). Moreover, high expression level of IGF2BP1 and IGF2BP3 has been detected in many human cancers, including glioma and lung adenocarcinoma. They have been correlated with invasiveness, aggressiveness and a poorer prognosis (38, 39). This analysis demonstrates good agreement with the predictions from the co-expression.

**Figure 12:**
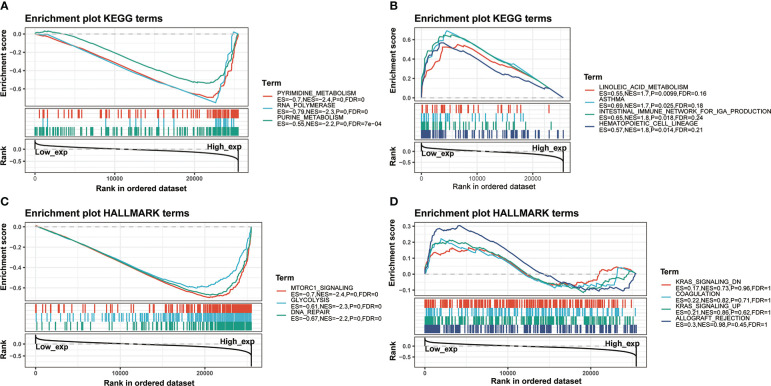
GSEA for samples with high IGF2BP3 expression and low expression. **(A)** The enriched gene sets in KEGG collection by the high IGF2BP3 expression sample. **(B)** The enriched gene sets in KEGG by samples with low IGF2BP3 expression. **(C)** Enriched gene sets in HALLMARK collection, the immunologic gene sets, by samples of high IGF2BP3 expression. **(D)** Enriched gene sets in HALLMARK by the low IGF2BP3 expression. Each line represented one particular gene set with unique color, and up-regulated genes located in the left approaching the origin of the coordinates, by contrast the down-regulated lay on the right of x-axis. Only gene sets with NOM p < 0.05 and FDR q < 0.25 were considered statistically significant. And only the leading-edge genes were displayed.

Furthermore, there was a significantly predictable link between IGF2BP3, LAPTM4A, and DHX57. GSEA was then conducted to determine the functional enrichment of high and low IGF2BP3 expression. The KEGG and HALLMARK analyses showed that IGF2BP3 was significantly linked to many immune-related signaling pathways **(** **Figure 12** **).**

### Drug sensitivity analysis of IGF2BP3

**Figure 13:**
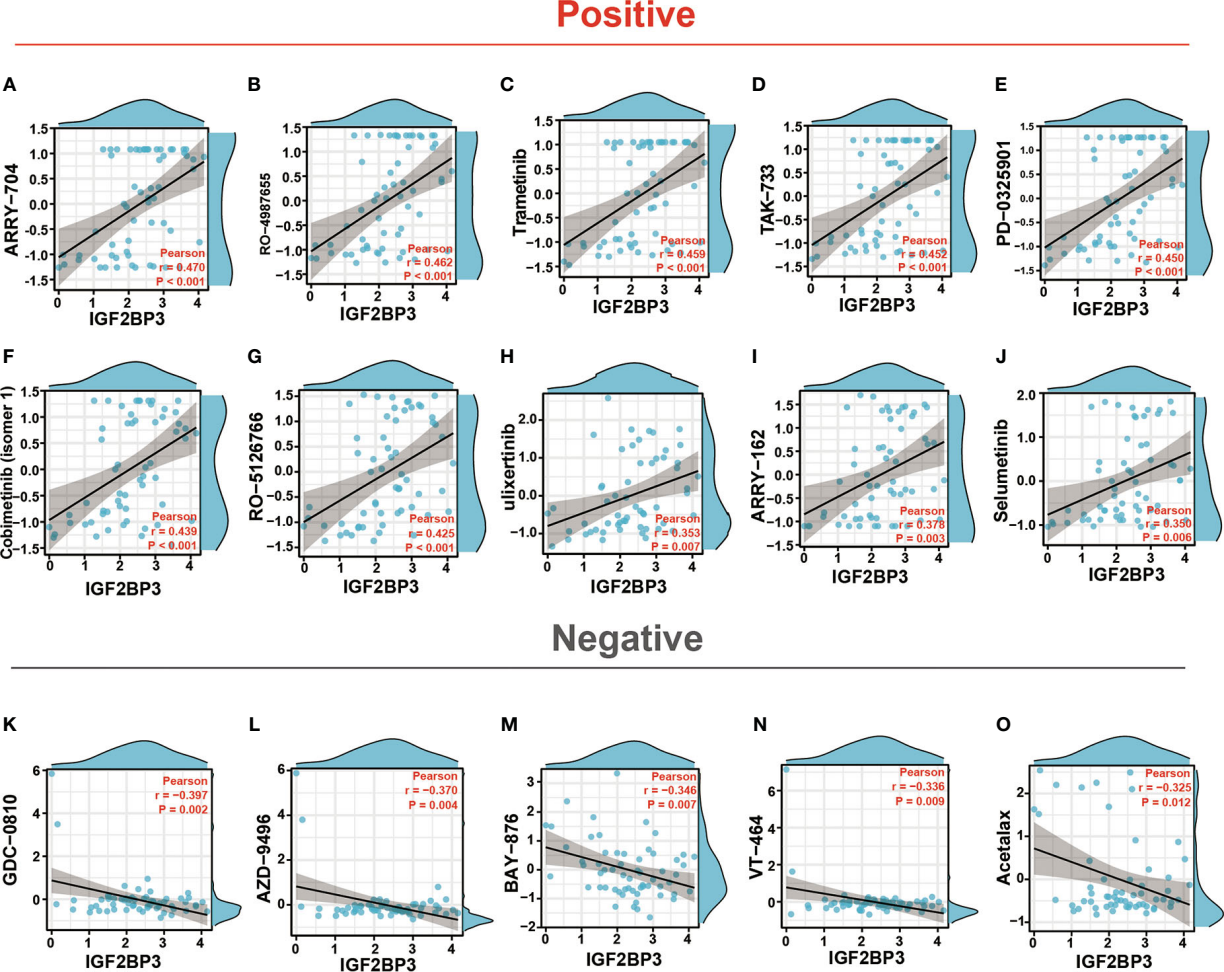
Drug sensitivity analysis of IGF2BP3. The expression of IGF2BP3 was associated with the sensitivity of ARRY-704 **(A)**, RO-4987655 **(B)**, Trametinib **(C)**, TAK-733 **(D)**, PD-0325901 **(E)**, Cobimetinib (isomer1) **(F)**, RO-5126766 **(G)**, Ulixertinib **(H)**, ARRY-162 **(I)**, Selumetinib **(J)**, GDC-0810 **(K)**, AZD-9496 **(L)**, BAY-876 **(M)**, VT-464 **(N)**, and Acetalax sensitivity **(O)**.

Enhancing drug sensitivity is crucial for preventing the drug resistance of cancer cells. We further investigated the potential correlation analysis between drug sensitivity and IGF2BP3 expression level accessed from the CellMiner database. Specifically, our results exhibited that IGF2BP3 had a significant and positive correlation with the clinical drug sensitivity of ARRY-704, RO-4987655, Trametinib, TAK-733, Mirdametinib, Cobimetinib, RO-5126766, Ulixertinib, ARRY-162, Selumetinib, *etc*(p < 0.01) (**Figures 13A–J**), while significant but negative associations with GDC-0810, AZD-9496, BAY-876, VT-464, and Acetalax sensitivity (p < 0.05) (**Figures 13K–O**). The data indicated that IGF2BP3 might be associated with chemoresistance of specific chemotherapeutic agents, such as Trametinib, Cobimetinib, ARRY-162 and Selumetinib, which were commonly used MEK inhibitors approved by the FDA for cancer therapy. These results established that IGF2BP3 was tightly linked to diverse drug sensitivity in different cancer cell lines and might serve as a promising therapeutic target for cancer immunotherapies.

### Validation of IGF2BP3 expression in glioma

**Figure 14:**
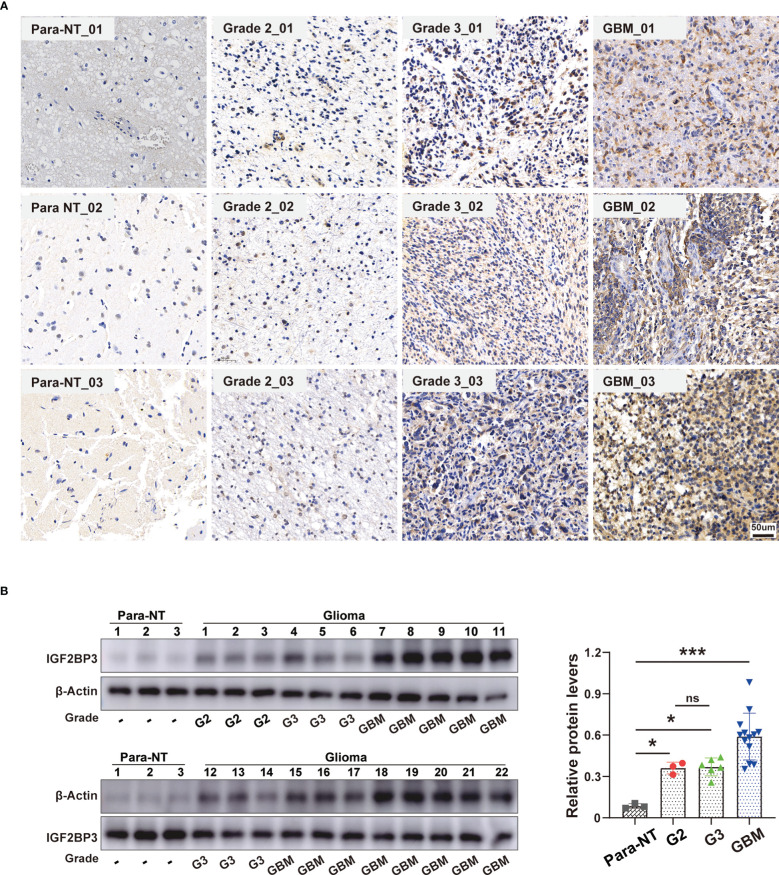
Validation of IGF2BP3 Expression in Glioma. **(A)** Representative immunohistochemical staining of IGF2BP3 expression in clinical glioma tissue and normal peritumor tissues. Scale bar=50 μm. **(B)** Western blot analysis of IGF2BP3 protein level in human glioma patient samples (grade 2 (n = 3), grade 3 (n = 6), grade GBM (n = 13)) and normal peritumor brain tissues (n = 3). β-actin was used as a loading control. All data are shown as the mean ± SD (at least three independent experiments). ns, no significance, *P <0.05, ***P < 0.001.

To further verify the pathophysiological roles of IGF2BP3, we applied experimental validation to determine its clinicopathological characteristics. We first evaluated the protein levels of IGF2BP3 in a series of clinical specimens, including nine glioma tissues (three specimens each from WHO grade 2,3,4 groups) and three peritumoral normal tissue using immunohistochemistry. The results showed that IGF2BP3 protein expression was significantly higher in glioma compared to normal tissue, especially in GBM **(** **Figure 14A** **)**. Western blot analysis further verified the expression of IGF2BP3 protein in glioma. We confirmed a similar expression trend at the protein level **(** **Figure 14B** **)**, suggesting that IGF2BP3 may be a potential molecular biomarker for the diagnosis and prognosis of glioma, especially GBM, which is expected to be a new therapeutic target for glioma.

## Discussion

IGF2BP3, also known as IMP3, a newly identified “reader” of m6A belonging to a highly conserved IGF2BP family (IGF2BP1/2/3) has been recognized to play an irreplaceable role in m6A modifications, mRNA stabilization, cell proliferation, and migration during the early stages of embryogenesis (40). Structurally, IGF2BP3 contains two N-terminal RNA recognition motifs (RRMs) and four C-terminal KH domains, which are critical for RNA-binding (5). As a m6A reader, IGF2BP3 was first reported in 1997 due to its high expression in pancreatic carcinoma (41). Subsequently, accumulative evidence has implied that the IGF2BP3 is post-transcriptionally active and plays a tumor-promoting role in various cancer types such as lung cancer (42), hepatocellular carcinoma (43), melanoma (44), and colorectal cancer (45), mainly by promoting tumor growth, invasion, metastasis, survival, and chemo-resistance (7–9).

In recent years, evidence has also suggested that IGF2BP3 might be a predictor of metastasis and clinical prognosis in different malignancies (46–50). For instance, IGF2BP3 could be a useful marker in predicting invasion in papillary biliary tumors (47). Moreover, both mRNA and protein levels of IGF2BP3 were remarkably up-regulated in cutaneous squamous cell carcinoma and IGF2BP3 was also identified as a novel therapeutic target for squamous cell carcinoma (48). Also, significant associations were found in colorectal cancer between IGF2BP3 positivity, poorer differentiation, and increased mortality, thus serving as a promising diagnostic biomarker for colorectal cancer in which higher expression indicates poorer prognosis (49). Further, a recent study revealed that m6A methylation regulators, IGF2BP2 and IGF2BP3, in particular, play essential roles in the malignant progression of glioma (50). However, upon reviewing the literature, there is no existing study comprehensively evaluating the significance of IGF2BP3 in pan-cancer on the whole scale. Of note, the pan-cancer analysis, which is of significant importance for understanding differences and similarities among different tumor types, can provide novel insights into cancer prevention and targeted therapy across cancer types. In recent years, there is increasing recognition of the value of a comprehensive pan-cancer analysis, which could potentially describe the essential roles of some driver mutations or genes in developing specific cancer types (51, 52).

In the present study, firstly, we used multiple databases to evaluate the expression level of IGF2BP3 across pan-cancer. The results showed that IGF2BP3 gene mRNA was highly expressed in most cancer types than in the normal samples, namely, GBM, GBMLGG, LGG, UCEC, CESC, LUAD, COAD, COADREAD, BRCA, ESCA, KIRP, KIPAN, STAD, HNSC, KIRC, LUSC, LIHC, SKCM, OV, PAAD, UCS, LAML, BLCA, ACC, KICH and CHOL, whereas low expression was detected in PRAD and THCA, which was consistent with previous studies in prostate and thyroid cancer **(** **Figure 1** **)** (53–55). IHC analysis from the HPA was in accordance with the IGF2BP3 mRNA level discrepancy and confirmed these results **(** **Figure 2** **)**. It is also noteworthy that either prostate or thyroid cancer has been thought to be a malignant disease-carrying a relatively favorable prognosis that can be diagnosed earlier (56). Additionally, according to prior studies, the oncofetal protein IGF2BP3 has been reported as a predominant cancer-specific marker differentiating benign from malignant lesions of pancreas and uterine cervix (57, 58), highly indicating that increased IGF2BP3 expression was associated with unfavorable prognosis among tumor tissues. These results demonstrated that IGF2BP3 could indeed promote cancer development and progression.

In addition, IGF2BP3 expression levels are tightly correlated with the immune subtypes of nine cancers, including CESC, LUAD, LUSC, LGG, COAD, STAD, BLCA, OV, and BRCA. Meanwhile, IGF2BP3 was significantly associated with diverse molecular subtypes in nine cancer types. For instance, IGF2BP3 was most highly expressed in the G-CIMP-low molecular isoforms in LGG and GBM, in the molecular subtype of classical in both LUSC and HNSC, and the molecular subtype of basal in BRCA. It is important to mention that IGF2BP3 is tightly associated with both immune and molecular subtypes in four types of cancers, including LGG, LUSC, BRCA, and COAD **(** **Figures 7**, **8** **)**.

Furthermore, we wondered whether IGF2BP3 played a critical role in cancer diagnosis and prognosis. ROC curve and Survival curve in pan-cancer plotted by Kaplan-Meier estimate revealed that IGF2BP3 had a certain accuracy (AUC>0.7) in predicting 24 cancer types, especially had a strong predictive power (AUC>0.9) in predicting LAML, GBM, UCS, LUSC, STAD, OV, CHOL, and ESCA. Moreover, IGF2BP3 was closely related to the OS, DSS, and PFI in GBMLGG, LGG, KIRP, KIRC, MESO, UVM, UCEC, and PAAD. Thus, IGF2BP3 might represent significant value as diagnostic and prognostic biomarkers in the individualized precision cancer therapy **(** **Figure 4** **)**.

Considering the important role of IGF2BP3 in gliomas, we further analyzed the role of IGF2BP3 in GBMLGG and identified significant correlations between IGF2BP3 expression levels and age, histological type and histological grade. Subsequently, we discovered that high expression of IGF2BP3 could cause a poorer OS, DSS, or PFI among a variety of clinical subgroups of GBMLGG. Since then, we confirmed WHO grade, age, IDH status, primary therapy outcome, gender, race, and IGF2BP3 expression level as independent indicators for the risk of OS, DSS, and PFI of GBMLGG through both univariate and multivariate Cox regression analyses **(** **Figure 6**; **Supplementary Figures 5**, **6)**.

IGF2BP3 may also have the potential as a therapeutic target for cancer treatment. Unlike chemotherapy, Immune checkpoint inhibitors help restore anti-tumor immune response, which has been shown to have a durable anti-tumor benefit in multiple cancers such as renal, melanoma, and lung cancers (59–61). Recently, increasing studies have reported that both TMB and MSI could be predictive biomarkers for identifying patients benefiting from immune checkpoint blockade therapies among multiple cancers (62–64), suggesting their potential response to immunotherapy. Moreover, the existing theory proved that an elevated TMB represented genomic instability associated with enhanced response to tumor immunotherapy (65, 66). In the present study, aberrant IGF2BP3 expression was found to be correlated with TMB in 14 cancer types, and MSI in 10 cancer types. The above correlation proved that IGF2BP3 was closely associated with the TME and might function as a promising biomarker for cancer immunotherapy in specific types of cancer. However, further experimental research is to prove its function **(** **Supplementary Figure 8** **)**.

Another principal finding of this study was the primary role of IGF2BP3 in cancer immunity. Recently, it has been well documented that the immune status of the tumor is closely associated with both critical components and tumor-infiltrating immune cell concentrations in TME (32, 67). ESTIMATE algorithm has been shown to be a favorable predictor of the levels of both tumor purity and immune infiltration in a variety of malignancies (32), including pancreatic cancer (68), colon cancer (69), and lung adenocarcinoma (70). Herein, using the TCGA database, we discovered that IGF2BP3 was significantly positively associated with the immune component of TME in 11 cancers, including BLCA, BRCA, COAD, KIRC, KIRP, LAML, LGG, PCPG, PRAD, READ, and UVM, negatively associated with the stromal component of TME in 4 cancers, including ACC, GBM, LUSC and UCEC **(** **Figure 9** **)**.

Following that, we found that IGF2BP3 expression was significantly positively correlated with the degree of B cell, neutrophil, CD8+, DC, and macrophage infiltration in LGG, PRAD, KIRC, THCA, BRCA, and GBMLGG. These cells are known to widely involved in both innate and adaptive immune responses (71, 72). Then, a close positive association between IGF2BP3 expression and several immune scores was detected in pan-cancer analysis. Thus, IGF2BP3 may represent a promising biomarker related to tumor immune cell infiltration, and it provides a possible regimen of immune-related therapies for many cancers.

Finally, in our current study of IGF2BP3 biological function, it was shown that IGF2BP3 presented significant participation in biological processes related to immune response and facilitated tumor development in various cancers **(** **Figure 12** **)**. A recent study uncovered better responses in patients with higher IGF2BP3 expression in anti-PD-1/PD-L1 therapy (22). Also, in the current study, IGF2BP3 was found to significantly correlate with classic immune checkpoint in human cancers, which remained one of the most successful immunotherapy strategies for multiple cancers. The results above implied the role of IGF2BP3 as a target in immunotherapy.

CellMiner is a website that provides genomics and pharmacology tools to identify drug patterns and transcripts in the NCI-60 cell line. Specifically, the CellMiner database contains 360 microRNAs, 22,379 genes, and 20,503 compounds incorporating 102 FDA-approved drugs (37). In our study, by searching the CellMiner database, we first explored the correlation between IGF2BP3 expression and anticancer drug sensitivity in detail. Results revealed that IGF2BP3 had a significantly positive association with most anticancer drugs, such as ARRY-704, RO-4987655, Trametinib, TAK-733, Cobimetinib, Mirdametinib, RO-5126766, AZD-0364, Ulixertinib, and Selumetinib **(** **Figure 13** **)**. Remarkably, the drugs mentioned here were all confirmed to be within the spectrum of inhibitors against the components (mainly MEK and ERK) of MAPK signaling pathway, which remains a key driver of tumor growth in human cancers (73). This finding also partly agreed with the previous work by Ramaswamy Suvasini et al. (74), while the latter established IGF2BP3 as a pivotal oncogenic factor expressed solely in the GBMs. Therefore, we deduced that IGF2BP3 might promote tumorigenesis by inhibiting positive regulators of the Raf/MEK/ERK pathway.

Although we have explored the pan-cancer role of IGF2BP3 from the perspective of bioinformatics in depth, we must acknowledge some limitations in the present study. To begin with, despite the conclusion that aberrant IGF2BP3 expression was associated with immune cell infiltration and prognosis of human cancers, we cannot definitively ascertain whether IGF2BP3 may exert functional effects on patient survival *via* an immune response. Therefore, the involvement of IGF2BP3 during immune regulation is still unclear and needs further investigation. Second, there is no clinical trial to evaluate the use of IGF2BP3-related therapeutic drugs in patients with pan-cancer. However, we have noted that a prognostic model containing eight genes, including IGF2BP3, for pediatric brain tumors has already been developed recently in a randomized controlled trial, which dramatically enhances the identification of those patients with a poorer prognosis by such gene signature (75). In the future, it is necessary to prospectively study the expression of IGF2BP3 and its significance in cancer immune infiltration, and to develop new drugs with higher anti-tumor activity targeting IGF2BP3.

## Conclusion

In conclusion, as far as we know, this is the first systematic study to elucidate the role of IGF2BP3 in pan-cancer from various angles, including its expression pattern, diagnosis, survival prognosis, genetic mutation, TMB, MSI, tumor immune microenvironment, relevant signaling pathways, and drug sensitivity. Based on our findings, IGF2BP3 may serve as a biomarker for the clinical detection of cancer. Our findings on the role of IGF2BP3 are prerequisites for clinical research and the practical application of IGF2BP3-based therapies.

## Data availability statement

The original contributions presented in the study are included in the article/**Supplementary Material**. Further inquiries can be directed to the corresponding authors.

## Ethics statement

The studies involving human participants were reviewed and approved by the Ethics Committee of Zhongshan Hospital of Fudan University (Shanghai, China). The patients/participants provided their written informed consent to participate in this study.

## Author contributions

PC and JX performed the statistical analysis and drew the pictures. PC performed experiments to vetify the expression of IGF2BP3 in glioma. JX, ZC, and SW performed the data analysis. PC: writing the article, critical revision of the article. XZ and TX contributed to the design of the study protocol. All authors contributed to the article and approved the submitted version.

## Glossary

**Table:**

RBP	RNA-binding protein
m6A	N6-methyladenosine
METTL3	methyltransferase-like protein 3
METTL14	methyltransferase-like 14
WTAP	Wilms-tumour associated protein
FTO	fat mass and obesity-associated protein
ALKBH5	AlkB homolog 5
TCGA	The Cancer Genome Atlas
GTEx	Genotype-Tissue Expression
CCLE	Cancer Cell Line Encyclopedia
HPA	Human Protein Atlas
DEGs	differentially expressed genes
TPM	transcripts per million reads
FC	fold-change
OS	overall survival
DSS	disease-specific survival
PFI	progression-free interval
WHO	World Health Organization
PD	progressive disease
SD	stable disease
PR	partial response
CR	complete response
TMB	tumor mutational burden
MSI	microsatellite instability
PPI	protein&ndash;protein interaction
KEGG	Kyoto Encyclopedia of Genes and Genomes
GSEA	Gene Set Enrichment Analysis
EMT	epithelial&ndash;mesenchymal transition
ACC	adrenocortical carcinoma
BLCA	bladder urothelial carcinoma
BRCA	breast invasive carcinoma
CESC	cervical squamous cell carcinoma and endocervical adenocarcinoma
CHOL	cholangiocarcinoma
COAD	colon adenocarcinoma
DLBC	lymphoid neoplasm diffuse large B-cell lymphoma
ESCA	esophageal carcinoma
GBM	glioblastoma multiforme
HNSC	head and neck squamous cell carcinoma
KICH	kidney chromophobe
KIRC	kidney renal clear cell carcinoma
KIRP	kidney renal papillary cell carcinoma
LAML	acute myeloid leukemia
LGG	brain lower grade glioma
LIHC	liver hepatocellular carcinoma
LUAD	lung adenocarcinoma
LUSC	lung squamous cell carcinoma
MESO	mesothelioma
OV	ovarian serous cystadenocarcinoma
PAAD	pancreatic adenocarcinoma
PCPG	pheochromocytoma and paraganglioma
PRAD	prostate adenocarcinoma
READ	rectum adenocarcinoma
SARC	sarcoma
SKCM	skin cutaneous melanoma
STAD	stomach adenocarcinoma
TGCT	testicular germ cell tumors
THCA	thyroid carcinoma
THYM	thymoma
UCEC	uterine corpus endometrial carcinoma
UCS	uterine carcinosarcoma
UVM	uveal melanoma

## References

[1] Dominissini D Moshitch-Moshkovitz S Schwartz S Salmon-Divon M Ungar L Osenberg S. Topology of the human and mouse m^6^A RNA methylomes revealed by m^6^A-seq. Nature (2012) 485(7397):201–06. doi: 10.1038/nature11112 22575960

[2] Wang X Zhao BS Roundtree IA Lu Z Han D Ma H. N(6)-methyladenosine modulates messenger RNA translation efficiency. Cell (2015) 161(6):1388–99. doi: 10.1016/j.cell.2015.05.014 PMC4825696 26046440

[3] He L Li H Wu A Peng Y Shu G Yin G. Functions of N6-methyladenosine and its role in cancer. Mol Cancer (2019) 18(1):176. doi: 10.1186/s12943-019-1109-9 31801551 PMC6892141

[4] Yang Y Hsu PJ Chen YS Yang YG. Dynamic transcriptomic m6A decoration: writers, erasers, readers and functions in RNA metabolism. Cell Res (2018) 28(6):616–24. doi: 10.1038/s41422-018-0040-8 PMC5993786 29789545

[5] Bell JL Wächter K Mühleck B Pazaitis N Köhn M Lederer M. Insulin-like growth factor 2 mRNA-binding proteins (IGF2BPs): post-transcriptional drivers of cancer progression? Cell Mol Life Sci (2013) 70(15):2657–75. doi: 10.1007/s00018-012-1186-z PMC3708292 23069990

[6] Huang H Weng H Sun W Qin X Shi H Wu H. Recognition of RNA N6-methyladenosine by IGF2BP proteins enhances mRNA stability and translation. Nat Cell Biol (2018) 20(3):285–95. doi: 10.1038/s41556-018-0045-z PMC5826585 29476152

[7] Mancarella C Scotlandi K. IGF2BP3 from physiology to cancer: Novel discoveries, unsolved issues, and future perspectives. Front Cell Dev Biol (2020) 7:363. doi: 10.3389/fcell.2019.00363 32010687 PMC6974587

[8] Nielsen J Christiansen J Lykke-Andersen J Johnsen AH Wewer UM Nielsen FC. A family of insulin-like growth factor II mRNA-binding proteins represses translation in late development. Mol Cell Biol (1999) 19(2):1262–70. doi: 10.1128/MCB.19.2.1262 PMC116055 9891060

[9] Lederer M Bley N Schleifer C Hüttelmaier S. The role of the oncofetal IGF2 mRNA-binding protein 3 (IGF2BP3) in cancer. Semin Cancer Biol (2014) 29:3–12. doi: 10.1016/j.semcancer.2014.07.006 25068994

[10] Wang Z Tong D Han C Zhao Z Wang X Jiang T. Blockade of miR-3614 maturation by IGF2BP3 increases TRIM25 expression and promotes breast cancer cell proliferation. EBioMedicine (2019) 41:357–69. doi: 10.1016/j.ebiom.2018.12.061 PMC6444029 30797711

[11] Endo I Amatya VJ Kushitani K Kambara T Nakagiri T Fujii Y. Insulin-like growth factor 2 mRNA binding protein 3 promotes cell proliferation of malignant mesothelioma cells by downregulating p27Kip1. Front Oncol (2022) 11:795467. doi: 10.3389/fonc.2021.795467 35127504 PMC8807558

[12] Li W Liu D Chang W Lu X Wang YL Wang H. Role of IGF2BP3 in trophoblast cell invasion and migration. Cell Death Dis (2014) 5(1):e1025. doi: 10.1038/cddis.2013.545 24457969 PMC4040666

[13] Hanniford D Ulloa-Morales A Karz A Berzoti-Coelho MG Moubarak RS Sánchez-Sendra B. Epigenetic silencing of CDR1as drives IGF2BP3-mediated melanoma invasion and metastasis. Cancer Cell (2020) 37(1):55–70. doi: 10.1016/j.ccell.2019.12.007 31935372 PMC7184928

[14] Zheng ZQ Li ZX Guan JL Liu X Li JY Chen Y. Long noncoding RNA TINCR-mediated regulation of acetyl-CoA metabolism promotes nasopharyngeal carcinoma progression and chemoresistance. Cancer Res (2020) 80(23):5174–88. doi: 10.1158/0008-5472.CAN-19-3626 33067266

[15] Yang Z Zhao F Gu X Feng L Xu M Li T. Binding of RNA m6A by IGF2BP3 triggers chemoresistance of HCT8 cells *via* upregulation of ABCB1. Am J Cancer Res (2021) 11(4):1428–45. PMC8085870 33948366

[16] Li Z Peng Y Li J Chen Z Chen F Tu J. N6-methyladenosine regulates glycolysis of cancer cells through PDK4. Nat Commun (2020) 11(1):2578. doi: 10.1038/s41467-020-16306-5 32444598 PMC7244544

[17] Li X Ma S Deng Y Yi P Yu J. Targeting the RNA m6A modification for cancer immunotherapy. Mol Cancer (2022) 21(1):76. doi: 10.1186/s12943-022-01558-0 35296338 PMC8924732

[18] Li Y Gu J Xu F Zhu Q Chen Y Ge D. Molecular characterization, biological function, tumor microenvironment association and clinical significance of m6A regulators in lung adenocarcinoma. Brief Bioinform (2021) 22(4):bbaa225. doi: 10.1093/bib/bbaa225 33003204

[19] Yang Z Wang T Wu D Min Z Tan J Yu B. RNA N6-methyladenosine reader IGF2BP3 regulates cell cycle and angiogenesis in colon cancer. J Exp Clin Cancer Res (2020) 39(1):203. doi: 10.1186/s13046-020-01714-8 32993738 PMC7523351

[20] Ding WB Wang MC Yu J Huang G Sun DP Liu L. HBV/Pregenomic RNA increases the stemness and promotes the development of HBV-related HCC through reciprocal regulation with insulin-like growth factor 2 mRNA-binding protein 3. Hepatology (2021) 74(3):1480–95. doi: 10.1002/hep.31850 33825218

[21] Yin H He H Shen X Zhao J Cao X Han S. miR-9-5p inhibits skeletal muscle satellite cell proliferation and differentiation by targeting IGF2BP3 through the IGF2-PI3K/Akt signaling pathway. Int J Mol Sci (2020) 21(5):1655. doi: 10.3390/ijms21051655 32121275 PMC7084337

[22] Wan W Ao X Chen Q Yu Y Ao L Xing W. METTL3/IGF2BP3 axis inhibits tumor immune surveillance by upregulating N6-methyladenosine modification of PD-L1 mRNA in breast cancer. Mol Cancer (2022) 21(1):60. doi: 10.1186/s12943-021-01447-y 35197058 PMC8864846

[23] Goldman MJ Craft B Hastie M Repečka K McDade F Kamath A. Visualizing and interpreting cancer genomics data *via* the xena platform. Nat Biotechnol (2020) 38(6):675–78. doi: 10.1038/s41587-020-0546-8 PMC7386072 32444850

[24] Barretina J Caponigro G Stransky N Venkatesan K Margolin AA Kim S. The cancer cell line encyclopedia enables predictive modelling of anticancer drug sensitivity. Nature (2012) 483(7391):603–07. doi: 10.1038/nature11003 PMC3320027 22460905

[25] Ru B Wong CN Tong Y Zhong JY Zhong SSW Wu WC. TISIDB: an integrated repository portal for tumor-immune system interactions. Bioinformatics (2019) 35(20):4200–2. doi: 10.1093/bioinformatics/btz210 30903160

[26] Cerami E Gao J Dogrusoz U Gross BE Sumer SO Aksoy BA. The cBio cancer genomics portal: an open platform for exploring multidimensional cancer genomics data. Cancer Discovery (2012) 2(5):401–04. doi: 10.1158/2159-8290.CD-12-0095 PMC3956037 22588877

[27] Gao J Aksoy BA Dogrusoz U Dresdner G Gross B Sumer SO. Integrative analysis of complex cancer genomics and clinical profiles using the cBioPortal. Sci Signal (2013) 6(269):pl1. doi: 10.1126/scisignal.2004088 23550210 PMC4160307

[28] Fancello L Gandini S Pelicci PG Mazzarella L. Tumor mutational burden quantification from targeted gene panels: major advancements and challenges. J Immunother Cancer (2019) 7(1):183. doi: 10.1186/s40425-019-0647-4 31307554 PMC6631597

[29] Boland CR Goel A. Microsatellite instability in colorectal cancer. Gastroenterology (2010) 138(6):2073–87. doi: 10.1053/j.gastro.2009.12.064 PMC3037515 20420947

[30] Rooney MS Shukla SA Wu CJ Getz G Hacohen N. Molecular and genetic properties of tumors associated with local immune cytolytic activity. Cell (2015) 160(1-2):48–61. doi: 10.1016/j.cell.2014.12.033 25594174 PMC4856474

[31] Thorsson V Gibbs DL Brown SD Wolf D Bortone DS Ou Yang TH. The immune landscape of cancer. Immunity (2018) 48(4):812–30.e14. doi: 10.1016/j.immuni.2018.03.023 29628290 PMC5982584

[32] Yoshihara K Shahmoradgoli M Martínez E Vegesna R Kim H Torres-Garcia W. Inferring tumour purity and stromal and immune cell admixture from expression data. Nat Commun (2013) 4:2612. doi: 10.1038/ncomms3612 24113773 PMC3826632

[33] Li T Fu J Zeng Z Cohen D Li J Chen Q. TIMER2.0 for analysis of tumor-infiltrating immune cells. Nucleic Acids Res (2020) 48(W1):W509–14. doi: 10.1093/nar/gkaa407 PMC7319575 32442275

[34] Yuan H Yan M Zhang G Liu W Deng C Liao G. CancerSEA: A cancer single-cell state atlas. Nucleic Acids Res (2019) 47(D1):D900–8. doi: 10.1093/nar/gky939 PMC6324047 30329142

[35] Warde-Farley D Donaldson SL Comes O Zuberi K Badrawi R Chao P. The GeneMANIA prediction server: biological network integration for gene prioritization and predicting gene function. Nucleic Acids Res (2010) 38(Web Server issue):W214–20. doi: 10.1093/nar/gkq537 PMC2896186 20576703

[36] Franz M Rodriguez H Lopes C Zuberi K Montojo J Bader GD. GeneMANIA update 2018. Nucleic Acids Res (2018) 46(W1):W60–4. doi: 10.1093/nar/gky311 PMC6030815 29912392

[37] Reinhold WC Sunshine M Liu H Varma S Kohn KW Morris J. CellMiner: a web-based suite of genomic and pharmacologic tools to explore transcript and drug patterns in the NCI-60 cell line set. Cancer Res (2012) 72(14):3499–511. doi: 10.1158/0008-5472.CAN-12-1370 PMC3399763 22802077

[38] Velasco MX Kosti A Penalva LOF Hernández G. The diverse roles of RNA-binding proteins in glioma development. Adv Exp Med Biol (2019) 1157:29–39. doi: 10.1007/978-3-030-19966-1_2 31342436

[39] Li W Li N Gao L You C. Integrated analysis of the roles and prognostic value of RNA binding proteins in lung adenocarcinoma. PeerJ (2020) 8:e8509. doi: 10.7717/peerj.8509 32071816 PMC7007976

[40] Mueller-Pillasch F Pohl B Wilda M Lacher U Beil M Wallrapp C. Expression of the highly conserved RNA binding protein KOC in embryogenesis. Mech Dev (1999) 88(1):95–9. doi: 10.1016/s0925-4773(99)00160-4 10525192

[41] Müeller-Pillasch F Lacher U Wallrapp C Micha A Zimmerhackl F Hameister H. Cloning of a gene highly overexpressed in cancer coding for a novel KH-domain containing protein. Oncogene (1997) 14(22):2729–33. doi: 10.1038/sj.onc.1201110 9178771

[42] Wang T Fan L Watanabe Y McNeill PD Moulton GG Bangur C. L523S, an RNA-binding protein as a potential therapeutic target for lung cancer. Br J Cancer (2003) 88(6):887–94. doi: 10.1038/sj.bjc.6600806 PMC2377073 12644826

[43] Jeng YM Chang CC Hu FC Chou HY Kao HL Wang TH. RNA-Binding protein insulin-like growth factor II mRNA-binding protein 3 expression promotes tumor invasion and predicts early recurrence and poor prognosis in hepatocellular carcinoma. Hepatology (2008) 48(4):1118–27. doi: 10.1002/hep.22459 18802962

[44] Pryor JG Bourne PA Yang Q Spaulding BO Scott GA Xu H. IMP-3 is a novel progression marker in malignant melanoma. Mod Pathol (2008) 21(4):431–37. doi: 10.1038/modpathol.3801016 18204432

[45] Li D Yan D Tang H Zhou C Fan J Li S. IMP3 is a novel prognostic marker that correlates with colon cancer progression and pathogenesis. Ann Surg Oncol (2009) 16(12):3499–506. doi: 10.1245/s10434-009-0648-5 19672661

[46] Zhang J Ji Q Jiao C Ren L Zhao Y Chen Y. IGF2BP3 as a potential tissue marker for the diagnosis of esophageal high-grade intraepithelial neoplasia. Onco Targets Ther (2017) 10:3861–66. doi: 10.2147/OTT.S141179 PMC5546816 28814885

[47] Sasaki M Sato Y. Insulin-like growth factor II mRNA-binding protein 3 (IMP3) is a marker that predicts presence of invasion in papillary biliary tumors. Hum Pathol (2017) 62:152–59. doi: 10.1016/j.humpath.2016.12.028 28089541

[48] Kanzaki A Kudo M Ansai S Peng WX Ishino K Yamamoto T. Insulin-like growth factor 2 mRNA-binding protein-3 as a marker for distinguishing between cutaneous squamous cell carcinoma and keratoacanthoma. Int J Oncol (2016) 48(3):1007–15. doi: 10.3892/ijo.2016.3323 PMC4750532 26782292

[49] Lochhead P Imamura Y Morikawa T Kuchiba A Yamauchi M Liao X. Insulin-like growth factor 2 messenger RNA binding protein 3 (IGF2BP3) is a marker of unfavourable prognosis in colorectal cancer. Eur J Cancer (2012) 48(18):3405–13. doi: 10.1016/j.ejca.2012.06.021 PMC3613860 22840368

[50] Guan S He Y Su Y Zhou L. A risk signature consisting of eight m6A methylation regulators predicts the prognosis of glioma. Cell Mol Neurobiol (2022) 42(8):2733–43. doi: 10.1007/s10571-021-01135-x PMC11421626 34432221

[51] Hong W Gu Y Guan R Xie D Zhou H Yu M. Pan-cancer analysis of the CASP gene family in relation to survival, tumor-infiltrating immune cells and therapeutic targets. Genomics (2020) 112(6):4304–15. doi: 10.1016/j.ygeno.2020.07.026 32682809

[52] Zhu L Wu W Jiang S Yu S Yan Y Wang K. Pan-cancer analysis of the mitophagy-related protein PINK1 as a biomarker for the immunological and prognostic role. Front Oncol (2020) 10:569887. doi: 10.3389/fonc.2020.569887 33244455 PMC7683787

[53] Chromecki TF Cha EK Pummer K Scherr DS Tewari AK Sun M. Prognostic value of insulin-like growth factor II mRNA binding protein 3 in patients treated with radical prostatectomy. BJU Int (2012) 110(1):63–8. doi: 10.1111/j.1464-410X.2011.10703.x 22077633

[54] Zhou AG Owens CL Cosar EF Jiang Z. Clinical implications of current developments in genitourinary pathology. Arch Pathol Lab Med (2013) 137(7):887–93. doi: 10.5858/arpa.2012-0210-RA 23808460

[55] Liu X Wang P Teng X Zhang Z Song S. Comprehensive analysis of expression regulation for RNA m6A regulators with clinical significance in human cancers. Front Oncol (2021) 11:624395. doi: 10.3389/fonc.2021.624395 33718187 PMC7946859

[56] Ito Y Miyashiro I Ito H Hosono S Chihara D Nakata-Yamada K. Long-term survival and conditional survival of cancer patients in Japan using population-based cancer registry data. Cancer Sci (2014) 105(11):1480–86. doi: 10.1111/cas.12525 PMC4462379 25183551

[57] Yantiss RK Woda BA Fanger GR Kalos M Whalen GF Tada H. KOC (K homology domain containing protein overexpressed in cancer): a novel molecular marker that distinguishes between benign and malignant lesions of the pancreas. Am J Surg Pathol (2005) 29(2):188–95. doi: 10.1097/01.pas.0000149688.98333.54 15644775

[58] Li C Rock KL Woda BA Jiang Z Fraire AE Dresser K. IMP3 is a novel biomarker for adenocarcinoma *in situ* of the uterine cervix: an immunohistochemical study in comparison with p16(INK4a) expression. Mod Pathol (2007) 20(2):242–47. doi: 10.1038/modpathol.3800735 17192788

[59] Constantinidou A Alifieris C Trafalis DT. Targeting programmed cell death -1 (PD-1) and ligand (PD-L1): A new era in cancer active immunotherapy. Pharmacol Ther (2019) 194:84–106. doi: 10.1016/j.pharmthera.2018.09.008 30268773

[60] Chen EX Jonker DJ Loree JM Kennecke HF Berry SR Couture F. Effect of combined immune checkpoint inhibition vs best supportive care alone in patients with advanced colorectal cancer: The Canadian cancer trials group CO.26 study. JAMA Oncol (2020) 6(6):831–8. doi: 10.1001/jamaoncol.2020.0910 PMC7206536 32379280

[61] Samstein RM Lee CH Shoushtari AN Hellmann MD Shen R Janjigian YY. Tumor mutational load predicts survival after immunotherapy across multiple cancer types. Nat Genet (2019) 51(2):202–06. doi: 10.1038/s41588-018-0312-8 PMC6365097 30643254

[62] Riaz N Havel JJ Makarov V Desrichard A Urba WJ Sims JS. Tumor and microenvironment evolution during immunotherapy with nivolumab. Cell (2017) 171(4):934–49. doi: 10.1016/j.cell.2017.09.028 PMC5685550 29033130

[63] Le DT Durham JN Smith KN Wang H Bartlett BR Aulakh LK. Mismatch repair deficiency predicts response of solid tumors to PD-1 blockade. Science (2017) 357(6349):409–13. doi: 10.1126/science.aan6733 PMC5576142 28596308

[64] Rizvi NA Hellmann MD Snyder A Kvistborg P Makarov V Havel JJ. Cancer immunology. mutational landscape determines sensitivity to PD-1 blockade in non-small cell lung cancer. Science (2015) 348(6230):124–28. doi: 10.1126/science.aaa1348 PMC4993154 25765070

[65] Sabari JK Leonardi GC Shu CA Umeton R Montecalvo J Ni A. PD-L1 expression, tumor mutational burden, and response to immunotherapy in patients with MET exon 14 altered lung cancers. Ann Oncol (2018) 29(10):2085–91. doi: 10.1093/annonc/mdy334 PMC6225900 30165371

[66] Li R Han D Shi J Han Y Tan P Zhang R. Choosing tumor mutational burden wisely for immunotherapy: A hard road to explore. Biochim Biophys Acta Rev Cancer (2020) 1874(2):188420. doi: 10.1016/j.bbcan.2020.188420 32828886

[67] Shiao SL Chu GC Chung LW. Regulation of prostate cancer progression by the tumor microenvironment. Cancer Lett (2016) 380(1):340–48. doi: 10.1016/j.canlet.2015.12.022 PMC5317350 26828013

[68] Bailey P Chang DK Nones K Johns AL Patch AM Gingras MC. Genomic analyses identify molecular subtypes of pancreatic cancer. Nature (2016) 531(7592):47–52. doi: 10.1038/nature16965 26909576

[69] Pagès F Mlecnik B Marliot F Bindea G Ou FS Bifulco C. International validation of the consensus immunoscore for the classification of colon cancer: a prognostic and accuracy study. Lancet (2018) 391(10135):2128–39. doi: 10.1016/S0140-6736(18)30789-X 29754777

[70] Wu J Li L Zhang H Zhao Y Zhang H Wu S. A risk model developed based on tumor microenvironment predicts overall survival and associates with tumor immunity of patients with lung adenocarcinoma. Oncogene (2021) 40(26):4413–24. doi: 10.1038/s41388-021-01853-y 34108619

[71] van der Leun AM Thommen DS Schumacher TN. CD8+ T cell states in human cancer: insights from single-cell analysis. Nat Rev Cancer (2020) 20(4):218–32. doi: 10.1038/s41568-019-0235-4 PMC7115982 32024970

[72] Hinshaw DC Shevde LA. The tumor microenvironment innately modulates cancer progression. Cancer Res (2019) 79(18):4557–66. doi: 10.1158/0008-5472.CAN-18-3962 PMC6744958 31350295

[73] Roberts PJ Der CJ. Targeting the raf-MEK-ERK mitogen-activated protein kinase cascade for the treatment of cancer. Oncogene (2007) 26(22):3291–310. doi: 10.1038/sj.onc.1210422 17496923

[74] Suvasini R Shruti B Thota B Shinde SV Friedmann-Morvinski D Nawaz Z. Insulin growth factor-2 binding protein 3 (IGF2BP3) is a glioblastoma-specific marker that activates phosphatidylinositol 3-kinase/mitogen-activated protein kinase (PI3K/MAPK) pathways by modulating IGF-2. J Biol Chem (2011) 286(29):25882–90. doi: 10.1074/jbc.M110.178012 PMC3138258 21613208

[75] Wang Y Zhou C Luo H Cao J Ma C Cheng L. Prognostic implications of immune-related eight-gene signature in pediatric brain tumors. Braz J Med Biol Res (2021) 54(7):e10612. doi: 10.1590/1414-431X2020e10612 34008756 PMC8130135

