# Supplementary material for: Multi-omics analysis of N6-methyladenosine reader IGF2BP3 as a promising biomarker in pan-cancer
Source: Front Immunol. 2023 Jan 25;14:1071675. doi: 10.3389/fimmu.2023.1071675 (PMC9905439; doi:10.3389/fimmu.2023.1071675)
Supplement: Supplementary file 1 [file DataSheet_1.zip › Supplementary_Material/Supplementary tables.docx]

| **Supplementary table 1.** Clinical characteristics of glioma patients. | | | |
| --- | --- | --- | --- |
| **Characteristic** | **Low expression of IGF2BP3** | **High expression of IGF2BP3** | ***p*** |
| ***n*** | **348** | **348** |  |
| **WHO grade, n (%)** |  |  | < 0.001 |
| G2 | 174 (27.4%) | 50 (7.9%) |  |
| G3 | 134 (21.1%) | 109 (17.2%) |  |
| G4 | 0 (0%) | 168 (26.5%) |  |
| **IDH status, n (%)** |  |  | < 0.001 |
| WT | 24 (3.5%) | 222 (32.4%) |  |
| Mut | 322 (46.9%) | 118 (17.2%) |  |
| **1p/19q codeletion, n (%)** |  |  | < 0.001 |
| codel | 159 (23.1%) | 12 (1.7%) |  |
| non-codel | 189 (27.4%) | 329 (47.8%) |  |
| **Primary therapy outcome, n (%)** |  |  | < 0.001 |
| PD | 51 (11%) | 61 (13.2%) |  |
| SD | 106 (22.9%) | 41 (8.9%) |  |
| PR | 46 (10%) | 18 (3.9%) |  |
| CR | 100 (21.6%) | 39 (8.4%) |  |
| **Age, n (%)** |  |  | < 0.001 |
| <=60 | 312 (44.8%) | 241 (34.6%) |  |
| >60 | 36 (5.2%) | 107 (15.4%) |  |
| **Histological type, n (%)** |  |  | < 0.001 |
| Astrocytoma | 96 (13.8%) | 99 (14.2%) |  |
| Glioblastoma | 0 (0%) | 168 (24.1%) |  |
| Oligoastrocytoma | 84 (12.1%) | 50 (7.2%) |  |
| Oligodendroglioma | 168 (24.1%) | 31 (4.5%) |  |
| Age, median (IQR) | 41 (33, 52) | 52 (37, 63) | < 0.001 |

**Supplemental table 2.** Details of diagnostic ROC for IGF2BP3 in pan-cancer.

| **Tumor type** | **Tumor(n)** | **Normal(n)** | **AUC(CI)** | **cut-off** | **sensitivity** | **specificity** | **Positive predictive value** | **Negative predictive value** | YI |
| --- | --- | --- | --- | --- | --- | --- | --- | --- | --- |
| ACC | **77** | 128 | 0.681(0.604-0.758) | 0.233 | 0.519 | 0.797 | 0.606 | 0.734 | 0.316 |
| BLCA | 407 | 28 | 0.747 (0.681-0.813) | 0.610 | 1.000 | 0.505 | 0.085 | 1.000 | 0.505 |
| BRCA | 1099 | 292 | 0.571 (0.536-0.605) | 0.520 | 0.238 | 0.914 | 0.913 | 0.242 | 0.153 |
| CESC | 306 | 13 | 0.881(0.984-1.000) | 0.516 | 0.755 | 0.846 | 0.991 | 0.128 | 0.601 |
| **CHOL** | **36** | **9** | **0.926 (0.844-1.000)** | **0.076** | **0.889** | **0.889** | **0.667** | **0.970** | **0.778** |
| COAD | 290 | 349 | 0.662 (0.616-0.708) | 0.963 | 0.479 | 0.943 | 0.874 | 0.685 | 0.422 |
| DLBC | 47 | 444 | 0.767 (0.713-0.821) | 0.599 | 0.830 | 0.680 | 0.215 | 0.974 | 0.510 |
| **ESCA** | **182** | **666** | **0.920(0.895-0.945)** | **2.517** | **0.758** | **0.935** | **0.762** | **0.934** | **0.694** |
| **GBM** | **166** | **1157** | **0.998(0.996-1.000)** | **0.860** | **0.982** | **0.990** | **0.931** | **0.997** | **0.972** |
| HNSC | 502 | 44 | 0.811(0.755-0.867) | 2.162 | 0.955 | 0.548 | 0.156 | 0.993 | 0.502 |
| KICH | 66 | 53 | 0.812(0.737-0.887) | 0.310 | 0.591 | 0.906 | 0.886 | 0.640 | 0.497 |
| KIRC | 531 | 100 | 0.801(0.756-0.846) | 0.245 | 0.699 | 0.830 | 0.956 | 0.342 | 0.529 |
| KIRP | 289 | 60 | 0.674(0.607-0.742) | 0.144 | 0.557 | 0.750 | 0.915 | 0.260 | 0.307 |
| **LAML** | **173** | **70** | **1.000 (1.000-1.000)** | **4.738** | **1.000** | **1.000** | **1.000** | **1.000** | **1.000** |
| LGG | 523 | 1152 | 0.762(0.737-0.787) | 0.170 | 0.621 | 0.765 | 0.545 | 0.816 | 0.386 |
| LIHC | 374 | 50 | 0.813 (0.771-0.855) | 0.065 | 0.920 | 0.719 | 0.305 | 0.985 | 0.639 |
| LUAD | 535 | 59 | 0.787(0.748-0.826) | 0.797 | 0.966 | 0.624 | 0.221 | 0.994 | 0.590 |
| **LUSC** | **498** | **338** | **0.939 (0.922-0.956)** | **1.763** | **0.841** | **0.985** | **0.988** | **0.808** | **0.827** |
| OSCC | 329 | 32 | 0.872(0.822-0.923) | 1.152 | 0.906 | 0.702 | 0.228 | 0.987 | 0.608 |
| **OV** | **427** | **88** | **0.927(0.901-0.953)** | **0.480** | **0.801** | **0.886** | **0.972** | **0.479** | **0.687** |
| PAAD | 179 | 171 | 0.835(0.793-0.877) | 0.937 | 0.715 | 0.836 | 0.821 | 0.737 | 0.551 |
| PRAD | 496 | 152 | 0.630(0.577-0.683) | 0.131 | 0.778 | 0.461 | 0.825 | 0.389 | 0.239 |
| READ | 93 | 318 | 0.450(0.373-0.528) | 0.170 | 0.323 | 0.745 | 0.270 | 0.790 | 0.068 |
| SKCM | 469 | 813 | 0.706 (0.677-0.735) | 0.459 | 0.921 | 0.652 | 0.604 | 0.935 | 0.573 |
| **STAD** | **414** | **210** | **0.936 (0.917-0.955)** | **0.669** | **0.850** | **0.933** | **0.962** | **0.760** | **0.784** |
| TGCT | 154 | 165 | 0.477(0.410-0.543) | 2.381 | 0.273 | 0.873 | 0.667 | 0.562 | 0.145 |
| THCA | 512 | 338 | 0.608(0.571-0.646) | 0.406 | 0.477 | 0.710 | 0.713 | 0.472 | 0.187 |
| THYM | 119 | 446 | 0.747(0.697-0.797) | 0.118 | 0.681 | 0.713 | 0.388 | 0.893 | 0.394 |
| UCEC | 552 | 35 | 0.855(0.792-0.918) | 0.154 | 0.771 | 0.859 | 0.257 | 0.983 | 0.630 |
| **UCS** | **57** | **78** | **0.983 (0.961-1.000)** | **0.361** | **0.965** | **0.962** | **0.948** | **0.974** | **0.926** |

Abbreviations: ROC: Receiver Operator Characteristic curve; AUC: Area Under Curve; CI: Confidence Interval; YI: Youden’s indx.

| **Supplemental Table 3** Univariate and multivariate Cox regression analyses of clinical characteristics associated with DSS of glioma. | | | | | | |
| --- | --- | --- | --- | --- | --- | --- |
| **Characteristics** | **Total(N)** | **Univariate analysis** | |  | **Multivariate analysis** | |
|  |  | **Hazard ratio (95% CI)** | **P value** |  | **Hazard ratio (95% CI)** | **P value** |
| **Primary therapy outcome** | 457 |  |  |  |  |  |
| PD | 111 | Reference |  |  |  |  |
| SD | 144 | 0.372 (0.242-0.572) | **<0.001** |  | 0.280 (0.158-0.497) | **<0.001** |
| PR | 64 | 0.138 (0.056-0.343) | **<0.001** |  | 0.102 (0.031-0.337) | **<0.001** |
| CR | 138 | 0.116 (0.053-0.252) | **<0.001** |  | 0.147 (0.064-0.335) | **<0.001** |
| **Age** | 674 |  |  |  |  |  |
| <=60 | 541 | Reference |  |  |  |  |
| >60 | 133 | 4.500 (3.409-5.940) | **<0.001** |  | 4.255 (2.470-7.331) | **<0.001** |
| **IGF2BP3** | 674 | 1.807 (1.672-1.952) | **<0.001** |  | 1.523 (1.241-1.868) | **<0.001** |

| **Supplemental table 4** Univariate and multivariate Cox regression analyses of clinical characteristics associated with PFI of glioma. | | | | | | |
| --- | --- | --- | --- | --- | --- | --- |
| **Characteristics** | **Total(N)** | **Univariate analysis** | |  | **Multivariate analysis** | |
|  |  | **Hazard ratio (95% CI)** | **P value** |  | **Hazard ratio (95% CI)** | **P value** |
| **Primary therapy outcome** | 461 |  |  |  |  |  |
| PD | 112 | Reference |  |  |  |  |
| SD | 147 | 0.253 (0.178-0.358) | **<0.001** |  | 0.262 (0.175-0.394) | **<0.001** |
| PR | 64 | 0.226 (0.137-0.372) | **<0.001** |  | 0.204 (0.108-0.385) | **<0.001** |
| CR | 138 | 0.160 (0.104-0.246) | **<0.001** |  | 0.179 (0.110-0.293) | **<0.001** |
| **IDH status** | 685 |  |  |  |  |  |
| WT | 246 | Reference |  |  |  |  |
| Mut | 439 | 0.151 (0.119-0.191) | **<0.001** |  | 0.500 (0.308-0.811) | **0.005** |
| **Age** | 695 |  |  |  |  |  |
| <=60 | 552 | Reference |  |  |  |  |
| >60 | 143 | 2.873 (2.268-3.640) | **<0.001** |  | 2.073 (1.363-3.154) | **<0.001** |
| **IGF2BP3** | 695 | 1.600 (1.497-1.710) | **<0.001** |  | 1.225 (1.040-1.444) | **0.015** |
