# Supplementary figures and images for: Multi-omics analysis of N6-methyladenosine reader IGF2BP3 as a promising biomarker in pan-cancer
Source: Front Immunol. 2023 Jan 25;14:1071675. doi: 10.3389/fimmu.2023.1071675 (PMC9905439; doi:10.3389/fimmu.2023.1071675)

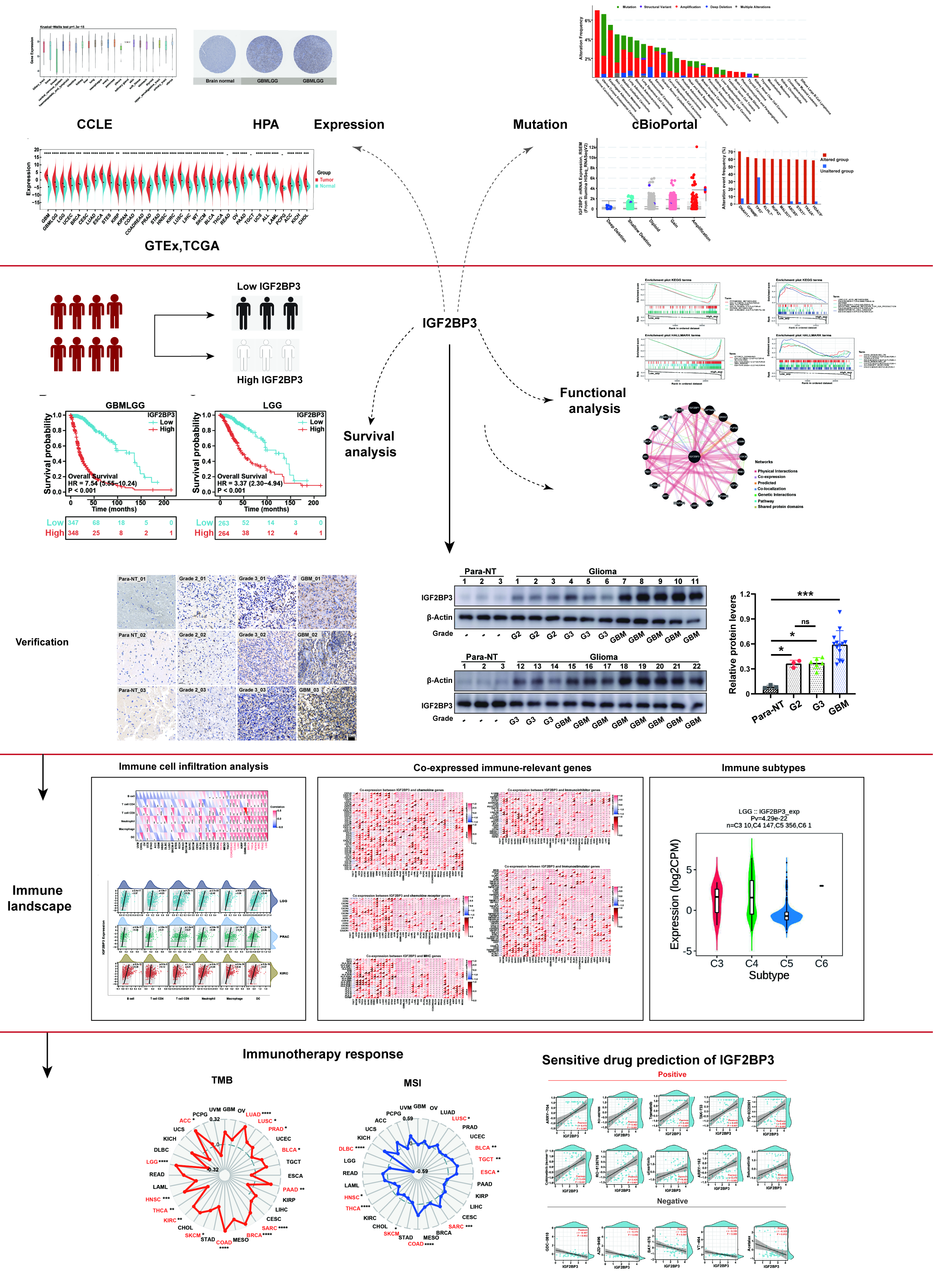

Supplement: Supplementary file 1 [file DataSheet_1.zip › Supplementary_Material/Supplementary Figure 1.tif]

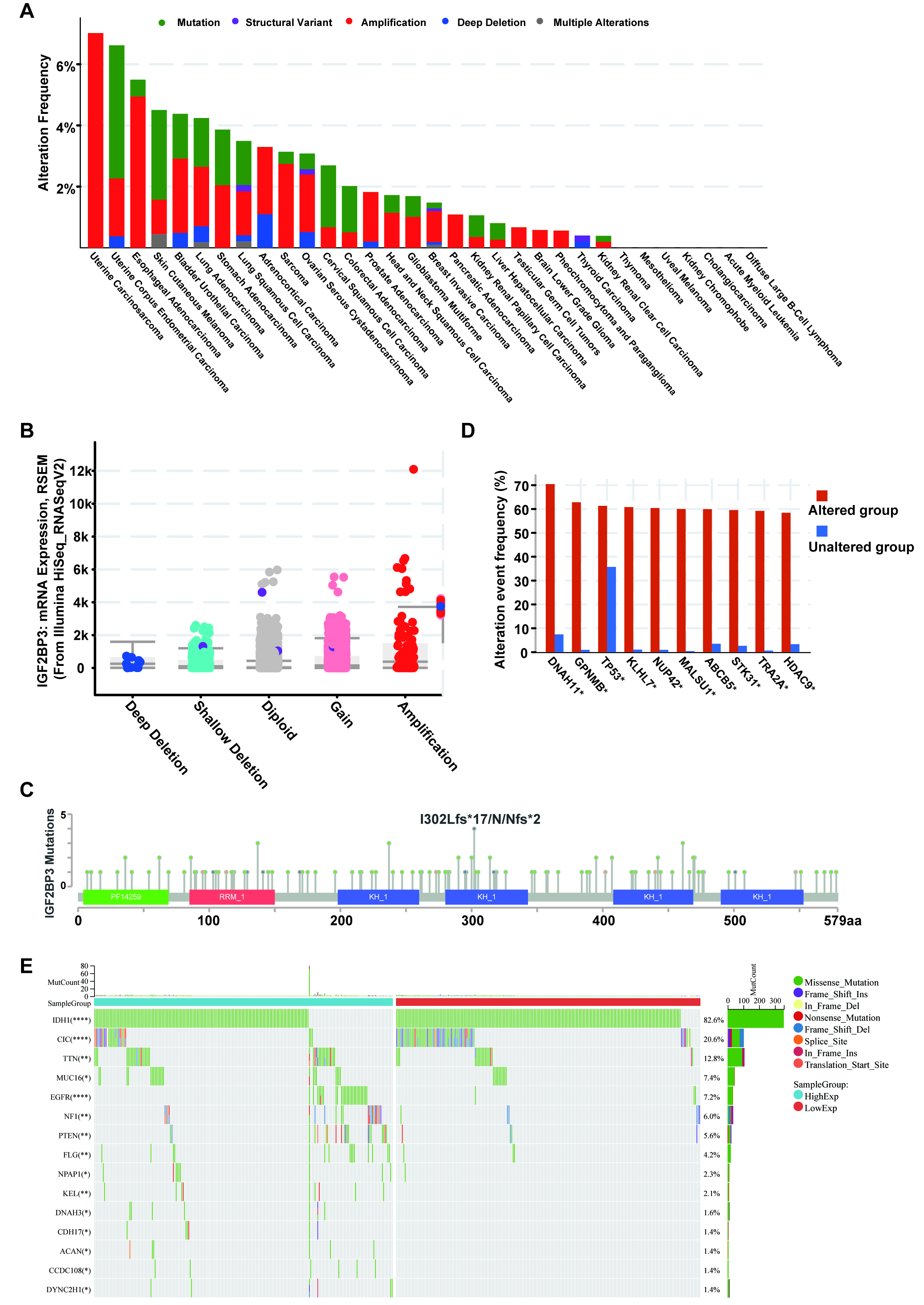

Supplement: Supplementary file 1 [file DataSheet_1.zip › Supplementary_Material/Supplementary Figure 2.tif]

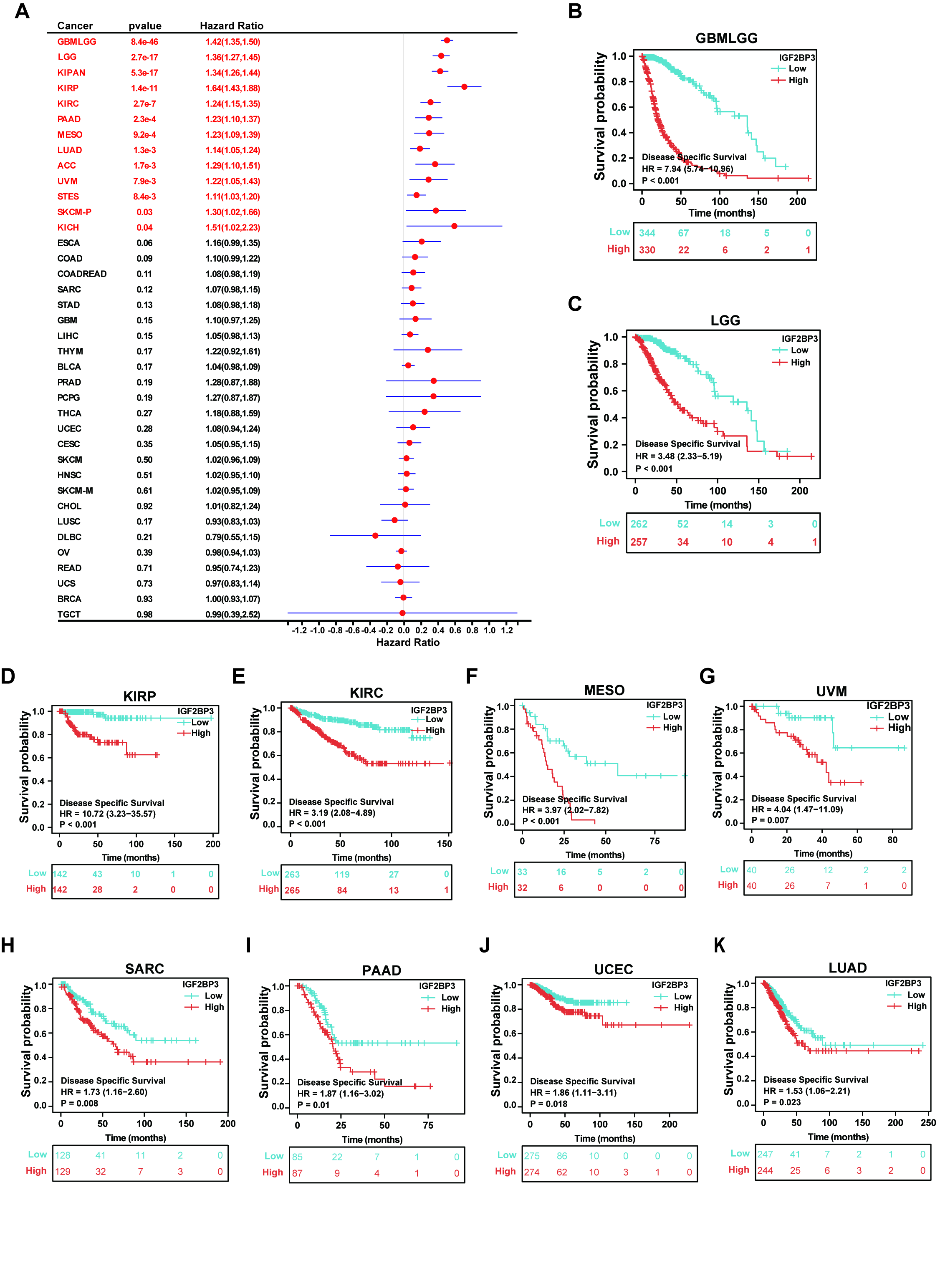

Supplement: Supplementary file 1 [file DataSheet_1.zip › Supplementary_Material/Supplementary Figure 3.tif]

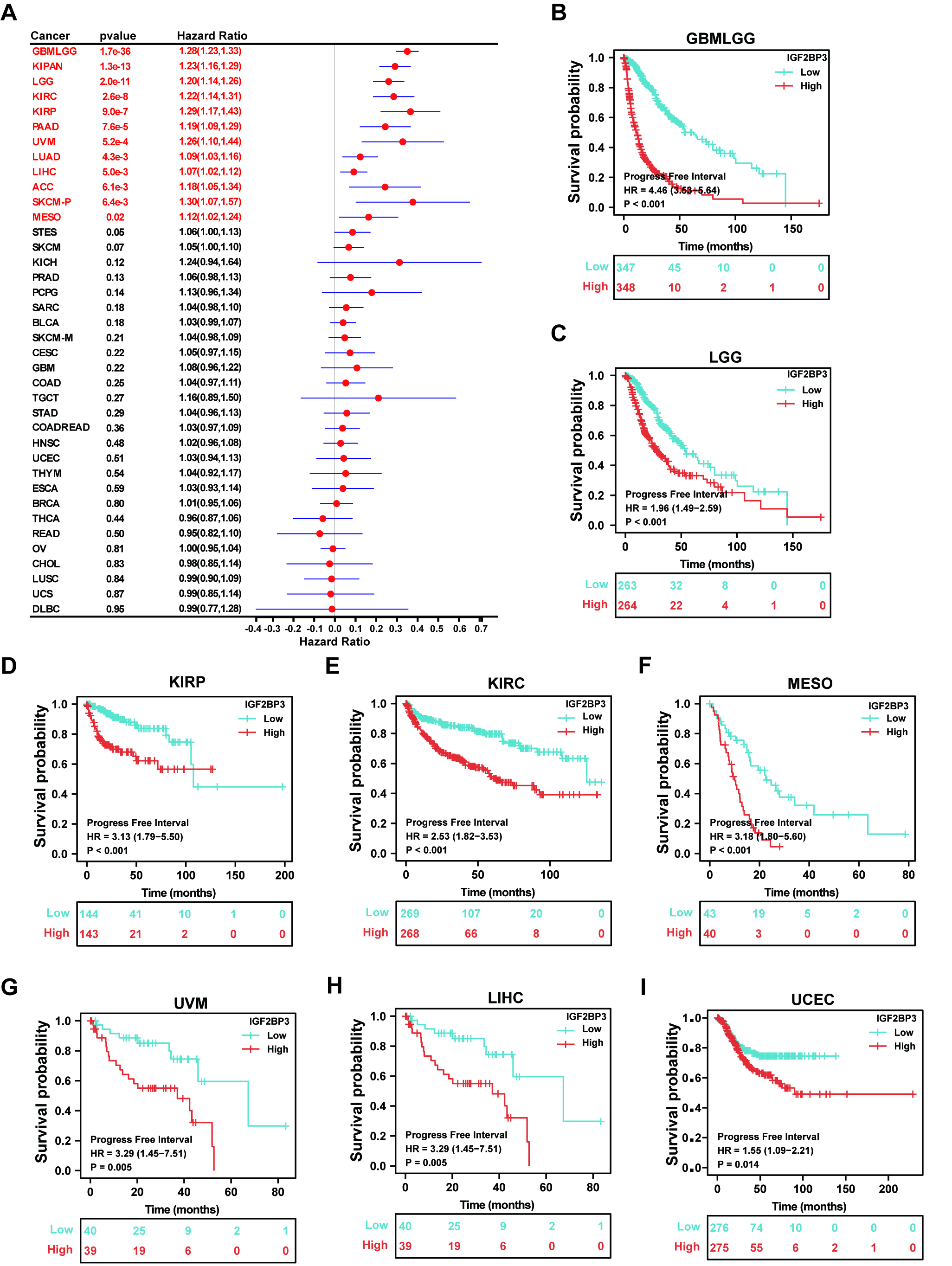

Supplement: Supplementary file 1 [file DataSheet_1.zip › Supplementary_Material/Supplementary Figure 4.tif]

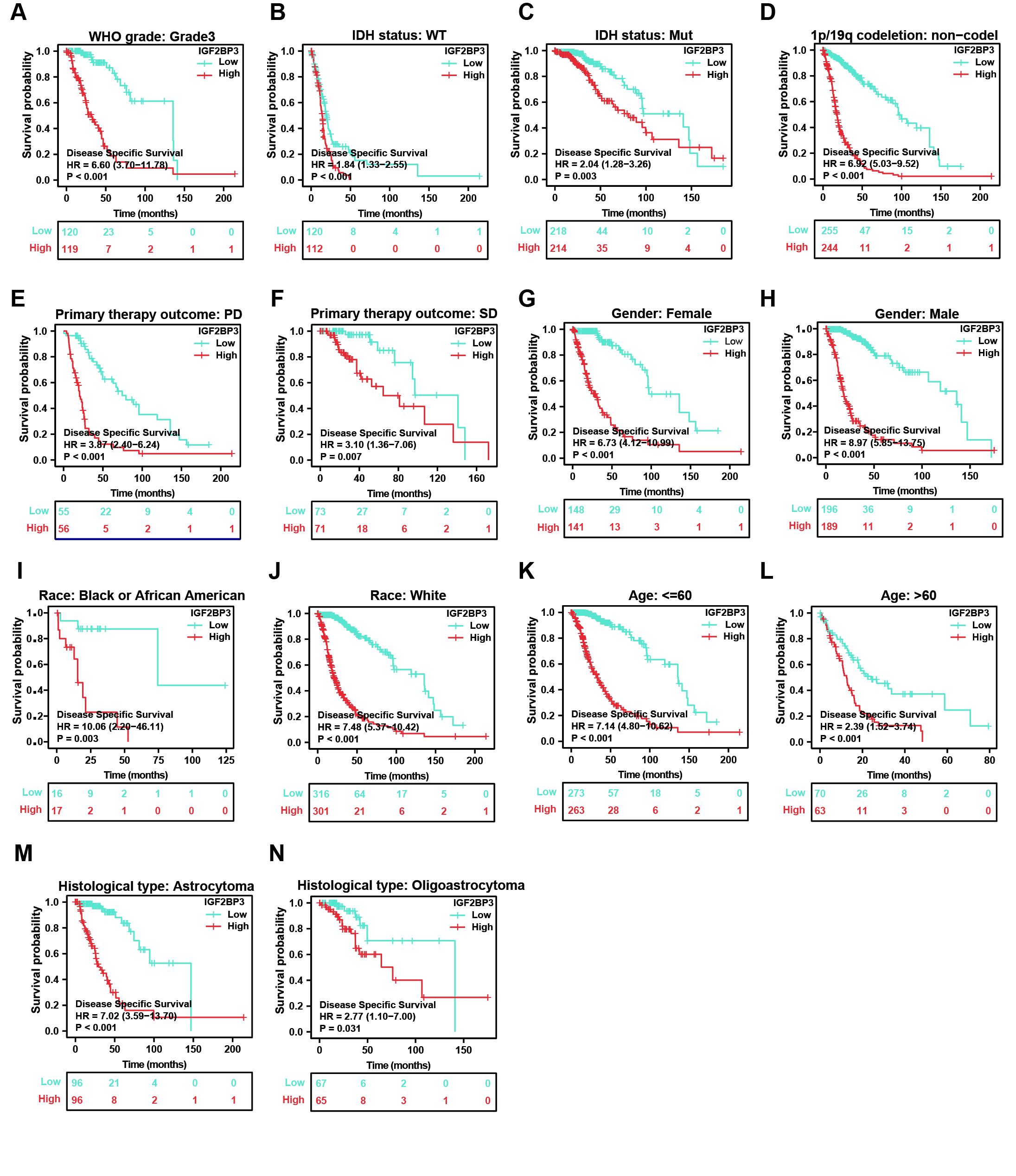

Supplement: Supplementary file 1 [file DataSheet_1.zip › Supplementary_Material/Supplementary Figure 5.tif]

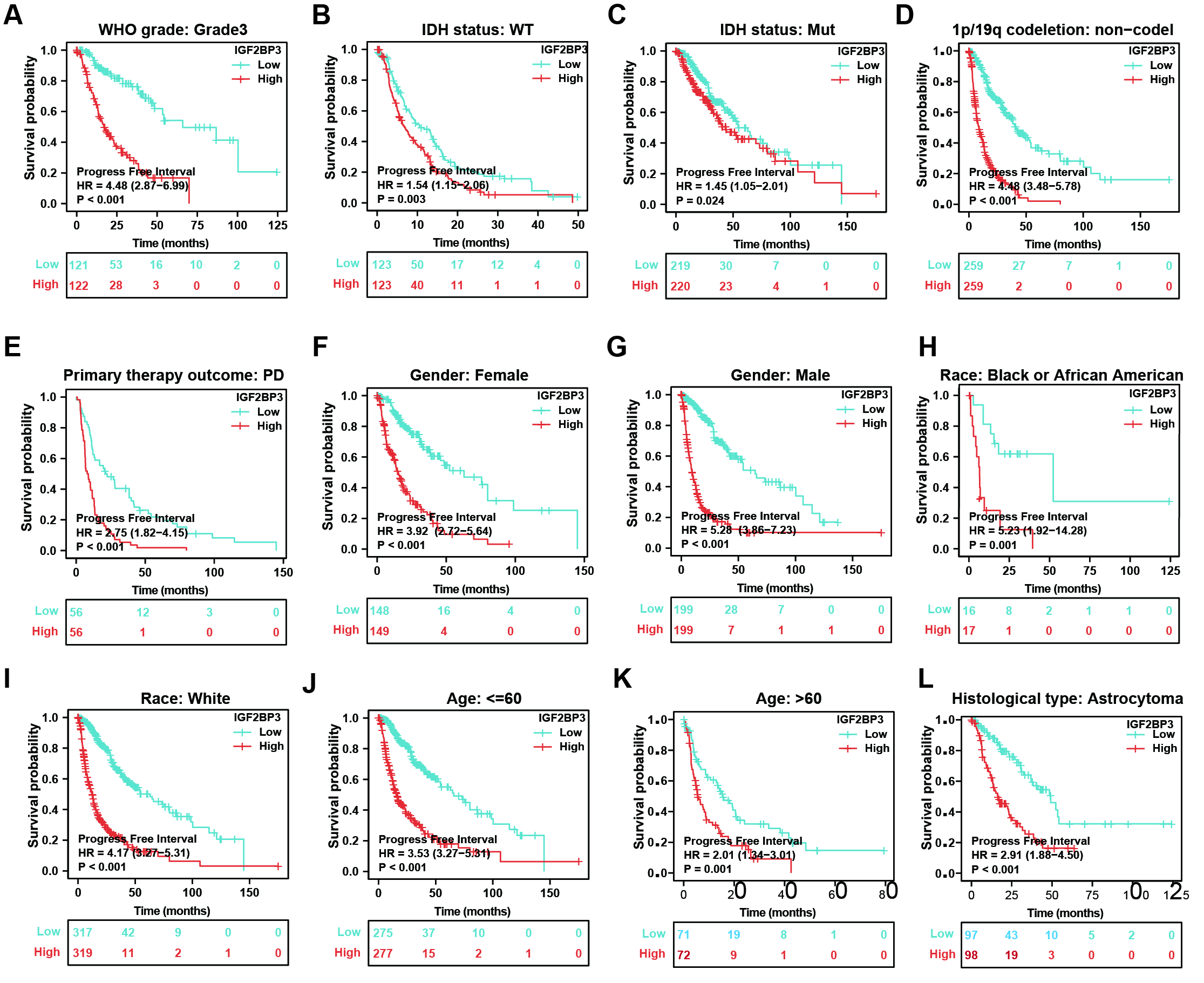

Supplement: Supplementary file 1 [file DataSheet_1.zip › Supplementary_Material/Supplementary Figure 6.tif]

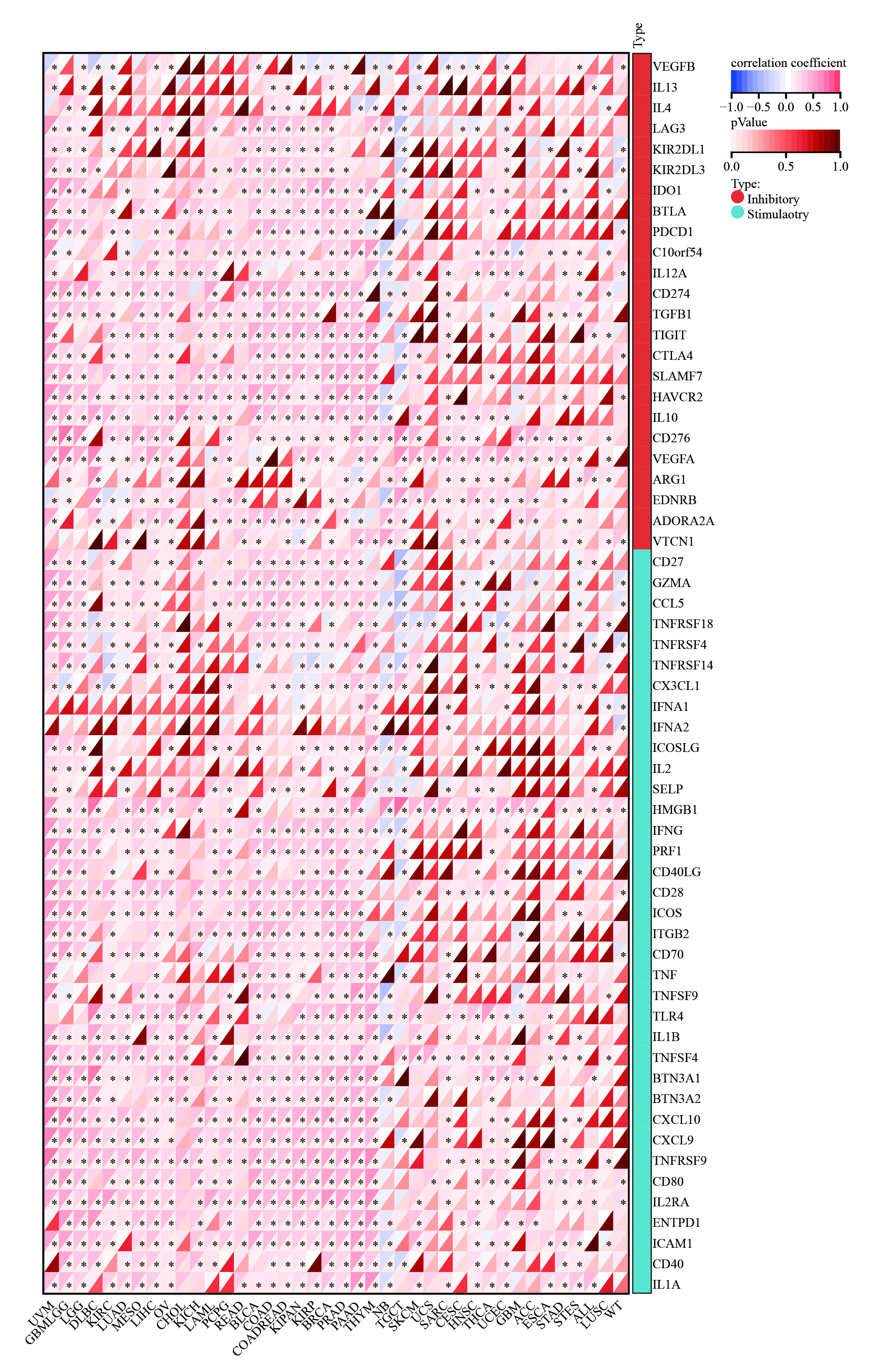

Supplement: Supplementary file 1 [file DataSheet_1.zip › Supplementary_Material/Supplementary Figure 7.tif]

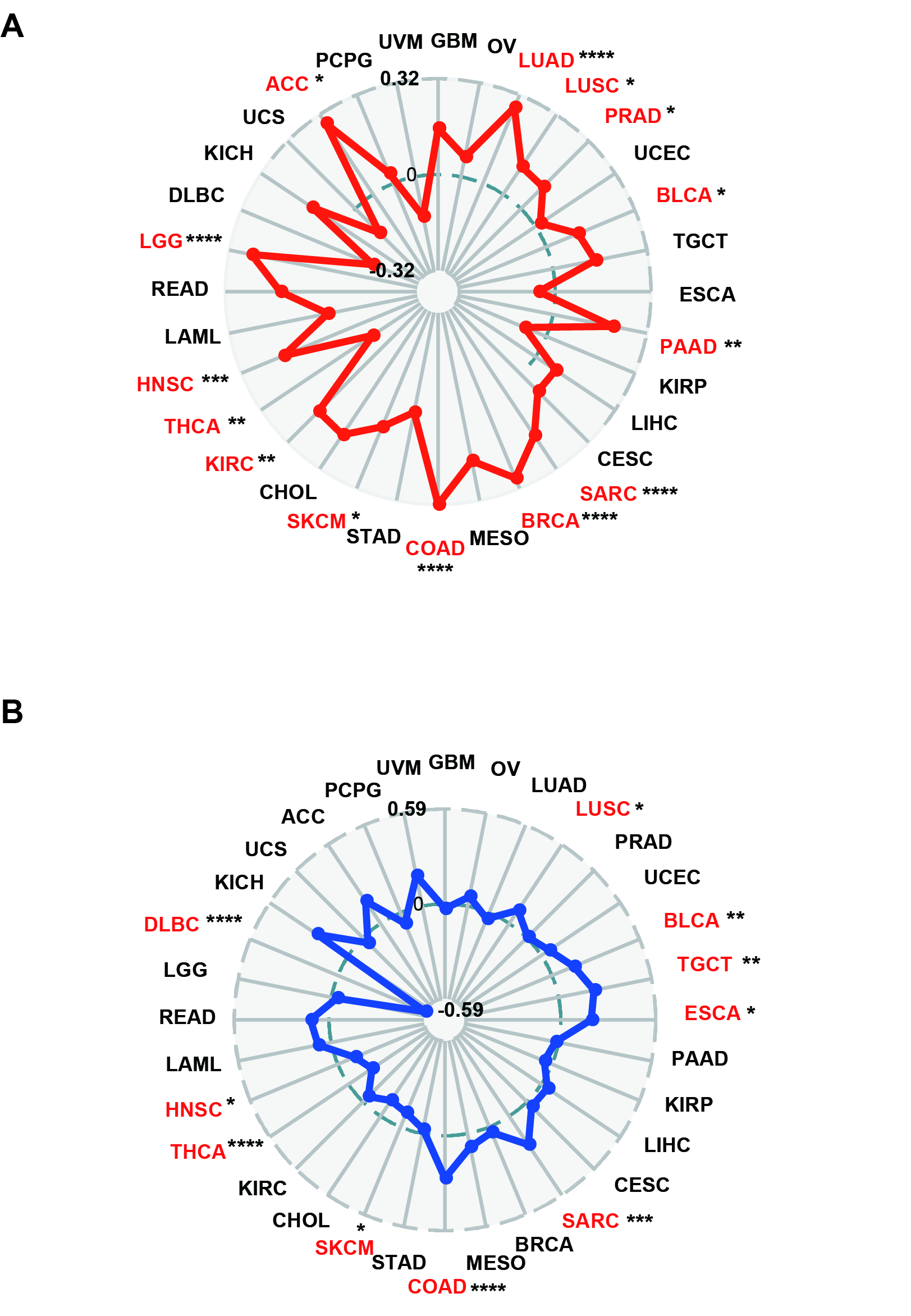

Supplement: Supplementary file 1 [file DataSheet_1.zip › Supplementary_Material/Supplementary Figure 8.tif]

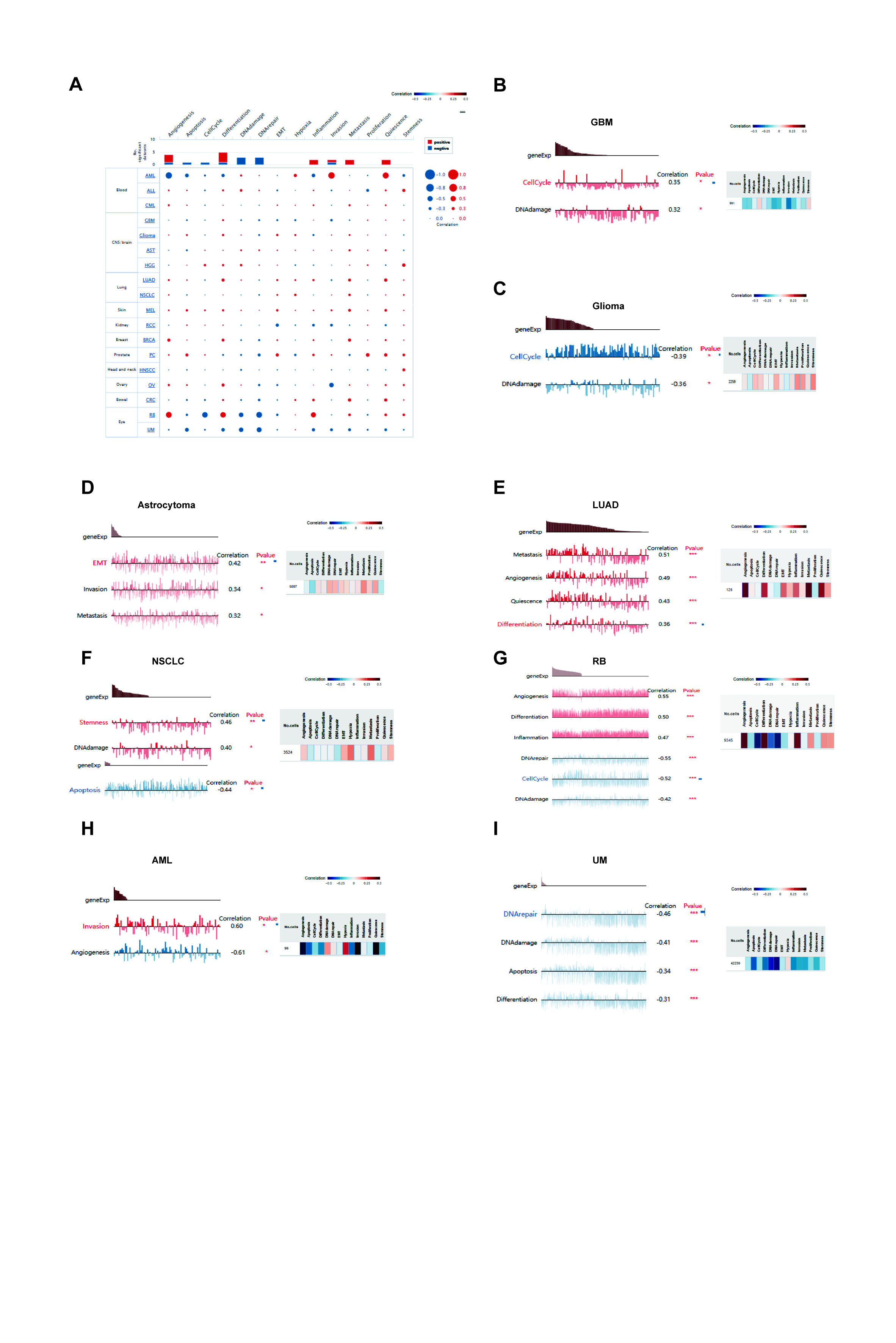

Supplement: Supplementary file 1 [file DataSheet_1.zip › Supplementary_Material/Supplementary Figures 9.tif]
